# Malformin C preferentially kills glioblastoma stem‐like cells via concerted induction of proteotoxic stress and autophagic flux blockade

**DOI:** 10.1002/1878-0261.13756

**Published:** 2024-10-27

**Authors:** Emma Phillips, Sizèd van Enk, Sara Kildgaard, Silja Schlue, Mona Göttmann, Victoria Jennings, Frederic Bethke, Gabriele Müller, Christel Herold‐Mende, Daniel Pastor‐Flores, Martin Schneider, Dominic Helm, Thomas Ostenfeld Larsen, Violaine Goidts

**Affiliations:** ^1^ Junior Research Group “Brain Tumor Translational Targets” German Cancer Research Center (DKFZ) Heidelberg Germany; ^2^ Department of Biotechnology and Biomedicine, Section for Microbial and Chemical Ecology, Natural Product Discovery Technical University of Denmark Copenhagen Denmark; ^3^ Division of Neurosurgical Research, Department of Neurosurgery University Hospital Heidelberg Germany; ^4^ Division of Redox Regulation German Cancer Research Center (DKFZ) Heidelberg Germany; ^5^ Proteomics Core Facility German Cancer Research Center (DKFZ) Heidelberg Germany

**Keywords:** autophagy, cancer, compound screen, glioblastoma, proteostasis, stem cells, unfolded protein response

## Abstract

Glioblastoma is a highly aggressive brain tumor for which there is no cure. The dire prognosis of this disease is largely attributable to a high level of heterogeneity, including the presence of a subpopulation of tumor‐initiating glioblastoma stem‐like cells (GSCs), which are refractory to chemo‐ and radiotherapy. Here, in an unbiased marine‐derived fungal extract screen, together with bioguided dereplication based on high‐resolution mass spectrometry, we identified malformin C to preferentially induce cell death in patient‐derived GSCs and explore the potential of this cyclic peptide as a therapeutic agent for glioblastoma. Malformin C significantly reduced tumor growth in an *in vivo* xenograft model of glioblastoma. Using transcriptomics and chemoproteomics, we found that malformin C binds to many proteins, leading to their aggregation, and rapidly induces the unfolded protein response, including autophagy, in GSCs. Crucially, chemical inhibition of translation using cycloheximide rescued malformin C‐induced cell death in GSCs, demonstrating that the proteotoxic effect of the compound is necessary for its cytotoxicity. At the same time, malformin C appears to accumulate in lysosomes, disrupting autophagic flux, and driving cells to death. Supporting this, malformin C synergizes with chloroquine, an inhibitor of autophagy. Strikingly, we observed that autophagic flux is differentially regulated in GSCs compared with normal astrocytes. The sensitivity of GSCs to malformin C highlights the relevance of proteostasis and autophagy as a therapeutic vulnerability in glioblastoma.

## Graphical abstract

In a fungal extract screen, malformin C was identified as a compound which selectively kills glioblastoma stem‐like cells while sparing normal cells. Malformin C induces proteotoxic stress and disrupts autophagy, exploiting a vulnerability in glioblastoma stem‐like cells. This discovery highlights the therapeutic potential of malformin C for treating this aggressive brain cancer.

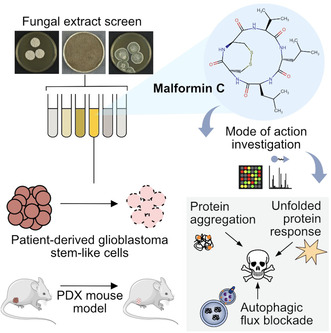

**Abbreviations**
7‐AAD: 7‐amino‐actinomycin
ATF4: activating transcription factor 4
Az‐Mal C: azide‐labeled malformin C
BafA1: bafilomycin A1
BMSCs: bone marrow stromal cells
BTIC: brain tumor‐initiating cells
CHX: cycloheximide
CLEAR: coordinated lysosomal expression and regulation
CQ: chloroquine
DMSO: dimethyl sulfoxide
Dox: doxycycline
eIF2α: eukaryotic translation initiation factor 2A
FDR: false discovery rate
GB: glioblastoma
GSC: glioblastoma stem‐like cell
GSEA: gene set enrichment analysis
GSH: glutathione
GSSG: reduced glutathione
H&E: hematoxylin and eosin
H2AX: H2A histone family member X
HFNSCs: human fetal neural stem cells
HSCs: hematopoietic stem cells
HSPCs: hematopoietic stem and progenitor cells
LC3B: microtubule‐associated proteins 1A/1B light chain 3B
Mal C: malformin C
NEM: *N*‐ethylmaleimide
NSG: NOD scid *gamma*
pAstro: primary human astrocytes
PERK: protein kinase R (PKR)‐like endoplasmic reticulum kinase
ROS: reactive oxygen species
SCARB1: Scavenger receptor class B member 1
TCGA: The Cancer Genome Atlas
TFRC: transferrin receptor protein 1
Tm: tunicamycin
TMRE: tetramethylrhodamine ethyl ester
TMZ: temozolomide
UHPLC‐DAD‐HRMS: ultra‐high‐performance liquid chromatography‐diode array detection high‐resolution mass spectrometry
UPR: unfolded protein response

## 1 Introduction

Glioblastoma (GB) is the most common newly diagnosed malignant brain tumor and is highly aggressive in nature. Even after surgery, radiation therapy, and chemotherapy, patient survival is poor, at around 15 months after diagnosis [1, 2]. Apart from the challenging location of the disease, this dire prognosis is largely attributable to a high level of heterogeneity, which inevitably leads to patient relapse even after promising initial responses to therapy. One major contributor to the heterogeneous nature of glioblastoma is the presence of a plastic differentiation hierarchy, with cancer stem cells at the apex [3, 4, 5]. Often referred to as brain tumor‐initiating cells (BTICs) or glioblastoma stem‐like cells (GSCs), these cells seem to persist after treatment, and their stem‐like properties enable them to repopulate the tumor, leading to patient relapse [6, 7, 8]. Therefore, targeting this subpopulation of glioblastoma cells could lead to more effective therapies for this deadly disease.

When considering small molecule cancer drugs that have been successful in the last 40 years, it is striking that around half of them are either natural products or natural product derivatives [9]. Having evolved to bind to a broad spectrum of proteins and other biological molecules, natural product structures tend to have characteristics of high chemical diversity and biochemical specificity, making them favorable as lead structures for drug discovery [10]. Filamentous fungi in particular are an invaluable source of diverse bioactive compounds, with single strains capable of producing hundreds of different secondary metabolites, depending on different environmental stimuli [11, 12]. Therefore, we performed bioactivity guided screening using a fungal library, which revealed malformin C to specifically reduce viability of GSCs compared with normal cells.

The malformins are cyclic pentapeptides containing three aliphatic amino acids, which vary between isoforms, and two adjacent cysteine residues which form a disulfide bond. A broad spectrum of biological properties has been reported for these molecules, including inducing the malformation of the stems and petioles of bean plants [13, 14], fibrinolytic activity [15, 16], and blocking the IL‐1 beta receptor [17]. Malformin A_1_ and malformin C have been investigated in the context of cancer research, having been shown to enhance bleomycin‐induced cell cycle arrest in Jurkat cells [18] and cell death in colon and ovarian cancer cell lines [19, 20]. Of particular interest, malformin A_1_ has been found to promote cell death through the induction of apoptosis, necrosis, and autophagy in prostate cancer cells by triggering oxidative stress, with the authors speculating that malformin A_1_ impairs the cellular function of glutathione to neutralize reactive oxygen species [21]. Up until now, the malformins have not been investigated in the context of brain tumors and cancer stem cells.

Using transcriptomic and chemoproteomic methods and state‐of‐the‐art phenotypic validation assays, in this work we decipher the mode of action for malformin C in GSCs, highlighting combined proteotoxic stress induction and autophagic flux blockade as a promising therapeutic modality for GB.

## 2 Materials and methods

### 2.1 Cell culture

GSC lines NCH421k, NCH441, NCH644, NCH711d, and NCH705 were derived from newly diagnosed GB patients who underwent surgical resection according to the research proposals approved by the Institutional Review Board at the Medical Faculty of Heidelberg (license: Neurosurgery brain tissue 005/2003; samples collected between 2003 and 2010 at Heidelberg University Hospital from patients who gave informed written consent) and performed in accordance to the Declaration of Helsinki. The cells were cultivated in serum‐free stem cell medium up to maximum passage 60 as previously described [22]. Human astrocytes originating from fetal cerebral cortex were purchased from ScienCell, Research Laboratories, Carlsbad, CA, USA (#1800) and cultured in DMEM (Sigma‐Aldrich, Steinheim, Germany) supplemented with 10% (v/v) FCS (Merck Millipore, Darmstadt, Germany), 1 mm sodium pyruvate, 1 × GlutaMAX, and 1 × N‐2 supplement (all Life Technologies, Darmstadt, Germany). Human fetal neural stem cells (HFNSCs) were kindly provided by Dr C Watts at the Department of Clinical Neurosciences, University of Cambridge. Tissue procurement was approved by the Cambridge Local Regional Ethics committee and in accordance with section 16(e) (ii) of the United Kingdom Human Tissue Act and the Declaration of Helsinki. They were established from 12‐ to 14‐week‐old human postmortem fetal tissue as described by Iovino et al [23]. The cells were cultured as neurospheres in neural stem cell medium (DMEM/F‐12 medium, 2% (v/v) B27 supplement, at 20 ng·mL^−1^ each of bFGF and EGF, 2.5 μg·mL^−1^ heparin). BJ cells (RRID: CVCL_3653) (human fibroblasts) were purchased from ATCC and cultivated in DMEM low glucose supplemented with 10% FCS. WTBL6 cells (mouse fibroblasts) were kindly provided by Gloria Lutzny at the Department of Haematology, Technical University of Munich, Germany and were cultured adherently Alpha MEM (Sigma‐Aldrich), supplemented with 1% (v/v) glutamate, 15% (v/v) FCS, 5% (v/v) horse serum, 1% (v/v) penicillin streptomycin, and 10 μm β‐mercaptoethanol. Hematopoietic stem and progenitor cells (HSPCs) were kindly provided by Andreas Trumpp at the division of Stem Cells and Cancer at the DKFZ, Heidelberg, Germany. Their extraction and enrichment were performed as described by Laurenti [24]. HEK293T (RRID: CVCL_0063), MDA‐MB‐231 (RRID: CVCL_0062), DU145 (RRID: CVCL_0105), SW480 (RRID: CVCL_0546), and U87 (RRID: CVCL_0022) cells were cultured in DMEM (Sigma‐Aldrich) supplemented with 10% (v/v) FCS (Merck Millipore). All cell lines were cultivated under standard conditions (37 °C, 95% humidity, 5% CO_2_, and 21% O_2_), authenticated using the in‐house service of the DKFZ, and tested biannually for mycoplasma at GATC.

### 2.2 Production of the fungal extract library

Fifteen strains of marine‐derived fungi belonging to *Aspergillus*, *Penicillium*, and *Stilbella* were cultivated on CYA and YES media plates and combined, extracted, and fractionated into 6–10 fractions to reveal the secondary metabolite profiles. Cultivation and dereplication of the strains have been previously described [25].

### 2.3 Identification of fungal strains

The various marine‐derived fungal strains belonging to *Aspergillus*, *Penicillium*, and *Stilbella* listed in Table S1 were 3‐point inoculated on CREA, CYA, YES, OAT, and MEA agar plates and incubated at 25 °C for 7 days in the dark. All fungal strains were from the collection at the Department of Systems Biology, DTU. The fungal species were confirmed based on their appearance on the different media and general morphology. *Aspergillus tubingensis* (IBT 28539) was isolated from a sea water trap set in the Labrador Sea, Greenland.

### 2.4 Cultivation of fungal strains and metabolite extraction

The marine‐derived fungal strains were 3‐point inoculated on eight plates of Czapek yeast extract agar (CYA) and yeast extract sucrose agar (YES) (four CYA and four YES) at 25 °C for 9 days in the dark. The extraction of the eight plates in total was achieved with 150 mL ethyl acetate containing 1% formic acid.

### 2.5 Metabolite isolation

The crude extracts were fractionated on a reversed phase C_18_ flash column (Sepra ZT, Isolute, 10 g) using an Isolera™ One automated flash system (Biotage, Uppsala, Sweden). The gradient used was acetonitrile and water buffered with 20 mm formic acid going from 15% to 100% acetonitrile over 28 min (12 mL·min^−1^). The fractions were automatically collected based on UV signal (210 and 254 nm), and each sample was divided into 9 to 12 fractions. For *Aspergillus tubingensis* (IBT 28539), nine flash fractions were automatically collected and further reduced into seven fractions based on the UV profile. Acetonitrile was of HPLC grade, and water was purified and deionized using a Millipore system through a 0.22 μm membrane filter (Milli‐Q water).

### 2.6 Purification of semi‐pure compounds from *A. tubingensis* (IBT 28539)

The isolation of semi‐pure compounds from the most bioactive fraction was performed using a semi‐preparative Gilson HPLC system (Middleton, WI, USA) with a 215 Liquid Handler, 819 Injection Module and a 172 DAD fully controlled with trilution lc software and achieved on a Luna II C18, 5 μm, 250 × 10 mm column (Phenomenex, Torrance, CA, USA) with a flow rate of 5 mL·min^−1^ using a linear gradient of 15% methanol in Milli‐Q water buffered with 50 ppm TFA going to 100% methanol in 20 min. Five semi‐pure compounds were automatically collected based on the UV absorption using the program ValveMate_falcon_abs_level and the absorption level 0.1 and a fraction collecting the remaining sample. The remaining sample was the bioactive one, and isolation of a sample including malformin A and C was conducted using a Waters 600 Controller (Milford, MA, USA) coupled to a Waters 996 Photodiode Array Detector and achieved on a Luna II C18, 5 μm, 250 × 10 mm column (Phenomenex, Torrance, CA, USA) with a flow rate of 5 mL·min^−1^ using a linear gradient 15% methanol in Milli‐Q water buffered with 50 ppm TFA going to 100% methanol in 20 min and manual collection for every 2 min.

### 2.7 Identification of Malformins A and C

The identification of malformins A and C was performed using ultra‐high‐performance liquid chromatography‐diode array detection‐quadrupole time of flight mass spectrometry (UHPLC‐DAD‐QTOF‐MS) with tandem HRMS fragmentation on an Agilent Infinity 1290 UHPLC system (Agilent Technologies, Santa Clara, CA, USA) equipped with a DAD and an Agilent 6550 iFunnel QTOFMS as previously described [25] and comparing the results obtained including retention times, HRMS, and MS/HRMS spectra with the spectra acquired using the commercial standards.

### 2.8 Fungal screen

The fungal screen was performed in nonbinding 96‐well white plates with clear bottoms (Greiner Bio‐One, Frickenhausen, Germany). A total of 20 000 NCH421k cells were seeded per well in 100 μL stem cell medium. Dried fractionated fungal extracts were dissolved in DMSO and 10, 2, 0.4, 0.1, and 0.025 μg were added per well. Positive (25 μm staurosporine) and negative (DMSO and medium only) controls were included on each plate. Cell viability was assessed after 24 and 48 h using the CellTiter‐Glo^®^ luminescent cell viability assay (Promega, Walldorf, Germany) according to the manufacturer's protocol. The signal obtained for each well was normalized to the average values of the 3 DMSO control wells on each plate. The data presented are from the 48 h time point. Active fractions were tested in subsequent replicate experiments.

### 2.9 Dose–response modeling

Cells were seeded in 96‐well plates at a density of 8500 cells per well. Adherent cells were seeded 1 day in advance to allow the cells to attach. Compound was applied at various concentrations in technical duplicate or triplicate, including vehicle (DMSO) control for normalization. Cell viability was assessed by the CellTiter‐Glo^®^ (Promega) luminescent cell viability assay. Incubation time varied depending on the compound and is stated in the figure legend. Data were normalized to the vehicle control, and four parameter logistic curves were fitted and IC_50_ values calculated using graphpad prism 9 software. For reducing malformin C, 100 μm malformin C in PBS was incubated with 400 μm DTT for 40 min and a further 40 min with 1.2 mm NEM at room temperature.

### 2.10 *In vivo* tumor propagation

NCH421k GSCs were transduced with lentiviral particles carrying the pLenti PGK V5‐LUC Neo plasmid, which was a gift from Eric Campeau (Addgene plasmid # 21471) [26] and transduced cells were selected using 400 μg·mL^−1^ G418. Six‐week‐old female NOD.Cg‐*Prkdc* ^*scid*^ *Il2rg* ^*tm1Wjl*^/SzJ (NOD‐SCIDγ) mice were obtained from the animal facility at the DKFZ. All animal experiments were performed according to animal welfare regulations and were approved by the responsible authorities (Regierungspräsidium Karlsruhe, approval number G289‐14). Animals were housed in a temperature‐controlled vivarium on a 12 h : 12 h light–dark cycle with *ad libitum* food and water, and all animals were monitored daily for health status and tumor‐related symptoms. Cells were treated with accutase and 5 × 10^6^ cells in 100 μL medium were mixed with 100 μL Matrigel (Corning, Kaiserslautern, Germany) and injected into the right flank of the mice. About 0.5 mg·kg^−1^ malformin C (or DMSO) was administered via *i.p*. injection three times per week. Tumor growth was monitored using an IVIS Lumina Preclinical *in vivo* imaging system and living image Software (version 2.50.1) (Perkin Elmer, Rodgau, Germany) and volume measurement using calipers once a week for 7 weeks, or until the tumor reached a volume of 500 mm^3^.

### 2.11 Pharmacokinetics

Pharmacokinetic (PK) parameters in plasma and blood brain barrier penetration after intravenous administration of malformin C were evaluated by Pharmacelsus GmbH. Nine adult male RjOrl : Swiss CD‐1 mice were injected *i.v*. with 0.5 mg·kg^−1^ malformin C. K‐EDTA plasma was obtained from the retrobulbar venous plexus under isoflurane anesthesia. The plasma samples were prepared within 45 min after collection, frozen at −20 °C, and stored at this temperature until processed for LC–MS analysis. For brain collection, mice were sacrificed by cervical dislocation, immediately after the last blood sample was obtained. Brains were dissected, frozen, and stored at −20 °C. PK analysis was performed using kinetica 5.0 Software, Allersberg, Germany. All experimental procedures were approved by and conducted in accordance with the regulations of the local Animal Welfare authorities (Landesamt für Gesundheit und Verbraucherschutz, Abteilung Lebensmittel‐und Veterinärwesen, Saarbrücken).

### 2.12 Immunohistochemistry

Mice were sacrificed by CO_2_ asphyxiation. Tumors were removed and perfused with 10% (w/v) formaldehyde. They were then cut in half, dehydrated in a STP 120 spin tissue processor, and embedded in paraffin. Sections of 4 μm thickness were cut and mounted onto glass slides, deparaffinized in xylene, and rehydrated in a descending series of alcohols. Slides were counterstained with hematoxylin and eosin and dehydrated. For Ki67 staining, the SuperVision 2 HRP‐polymer kit (DCS, PD000POL) was used according to the manufacturer's protocol. Slides were treated with anti‐Ki67 (1 : 2000, Abcam, Cambridge, UK, 15580) in antidiluent with background reducing components (Dako, Jena, Germany, S302) overnight at 4 °C. Images were taken using a Zeiss Axioplan 2 microscope, Oberkochen, Germany.

### 2.13 Caspase assay

A total of 5000 cells were seeded into white 96‐well plates in a volume of 50 μL medium. Malformin C or Az‐mal. C was added to the medium at the indicated concentrations and incubated for the indicated times. Caspase 3/7 activity was measured using the Caspase Glo assay (Promega), according to the manufacturer's protocol.

### 2.14 Apoptosis assay

A total of 200 000 GSCs were treated with malformin C for 48 h in 2 mL medium. Cells were dissociated with accutase and incubated with annexin V binding buffer containing 10% 7‐amino‐actinomycin (7‐AAD) and 10% annexin V‐PE (BD Biosciences) for 15 min at 4 °C. Annexin V and 7‐AAD‐positive cells were measured using a FACSCanto II and quantified with facsdiva Software (BD Biosciences, Heidelberg, Germany).

### 2.15 CD133 staining

Cells were dissociated with accutase and incubated for 20 min at 4 °C with CD133‐PE conjugated antibody (1 : 200, MACS, 130‐098‐826) (or IgG1‐PE isotype control) in 5% FCS/PBS. After 2 × washing in 5% FCS/PBS, cells were resuspended in 200 μL 5% FCS/PBS, transferred into FACS tubes, and analyzed using a FACSCanto^TM^II. Quantification followed using facsdiva Software.

### 2.16 Microarray analysis

RNA was extracted with RLT buffer and lysates were homogenized using QIAGEN QIAshredder columns. The QIAGEN RNeasy mini kit was used in accordance with the manufacturer's protocol. The Illumina TotalPrep RNA Amplification Kit (Life Technologies) was used to generate single‐stranded cRNA from input amounts of 200 ng total RNA. About 750 ng of cRNA were hybridized for 17 h at 58 °C on an Illumina Human HT‐12‐Microarray using the standard Illumina hybridization protocol. Gene expression microarrays were scanned using the Illumina iScan‐Scanner according to the standard Illumina scanning protocol. The data were normalized using quantile normalization with R using the function normalize.quantiles from Bioconductor package ‘preprocessCore’.

### 2.17 Gene set enrichment analysis and visualization

GSEA analysis was performed at www.broadinstitute.org/gsea. Genes were preranked based on the mean fold change upon malformin C treatment and analyzed for the enrichment of the GO signatures (c5.all.v7.0.symbols, 6539 signatures), as listed in the Molecular Signatures Database (http://www.broadinstitute.org/gsea/msigdb). Quantification of enrichment of the signature genes near the top of the ordered list of genes followed using a running‐sum statistic, resulting in an enrichment score. The FDR q‐value was used to set a significant threshold. The network of gene set interactions was constructed using cytoscape Consortium, San Diego, CA, USA (version 3.7.2).

### 2.18 Western blot

For analysis of protein levels, 200 000 cells were seeded in 2 mL medium in 6‐well plates and treated accordingly. Total protein was extracted, separated, transferred, and visualized as described previously [22]. For separation of proteins into soluble and insoluble fractions, a Triton X‐100 containing buffer was used to extract detergent‐soluble proteins, and insoluble aggregates were extracted using urea and benzonase as detailed in [27]. Primary antibodies against the listed proteins were used as follows: ATF4 (1 : 500, Cell Signaling, 11815S), LC3B (1 : 1000, Cell Signaling, 2775S) EIF2α (1 : 1000, Cell Signaling, Leiden, The Netherlands, 9722S), P‐EIF2α, ubiquitin (1 : 1000, Sigma, U‐0508), CHOP (1 : 500, Cell Signaling, 2895), ATP6V1H (1 : 1000, Protein Tech, Planegg, Germany, 26683‐1‐AP), TMX1 (1 : 1000, Protein Tech, 27489‐1‐AP), P‐p38 (1 : 1000, Cell Signaling, 4511), γH2Ax (1 : 300, Merck Millipore, 05‐636), SQLE (1 : 1000, Santa Cruz Biotechnology, Heidelberg, Germany, sc‐271651), TOMM40 (1 : 1000, Protein Tech, 18409‐1‐AP), PRD6X (1 : 1000, Protein Tech, 13585‐1‐AP), TFRC (1 : 1000, Invitrogen, Darmstadt, Germany, 13‐6800), β‐actin (1 : 5000, Cell Signaling; 3700S), and α‐tubulin (1 : 5000, Sigma, T9026). Horseradish peroxidase (HRP) coupled secondary antibodies (Cell Signaling; mouse 7076S, rabbit 7074S) were used at dilutions of 1 : 5000.

### 2.19 Detection of γH2AX

An optimized lysis protocol was developed for the detection of γH2AX by western blotting. Cells were lysed in 2 mm MgCl_2_, 62.5 mm Tris (pH 7.5) and 1% SDS supplemented with protease (Roche, Penzberg, Germany) and phosphatase inhibitors (PhosSTOP™, Sigma). About 1 μL of benzonase was added to each sample and incubation for 15 min at RT ensued before the reaction was stopped with 2 mm EDTA. Loading dye and reducing agent were added as for the standard lysates, but samples were not heated prior to loading on the gel.

### 2.20 Measurement of mitochondrial membrane potential

Mitochondrial membrane potential was measured using tetramethylrhodamine ethyl ester (TMRE) staining, which is readily sequestered in active mitochondria, and FACS analysis. A total of 100 000 cells singularized and were treated with malformin C in 12‐well plates. Positive control FCCP (20 μm) was added 40 min, and TMRE (200 nm) 30 min prior to analysis. Medium was changed to 0.2% BSA/PBS, and PE intensity was measured on a FACSCanto II and quantified with facsdiva Software (BD Biosciences).

### 2.21 Measurement of oxygen consumption

Oxygen consumption as an indication of rate of oxidative phosphorylation was measured using an Oxygraph‐2K (Oroboros Inc., Innsbruck, Austria) machine. NCH421k cells were singularized and resuspended in 2.2 mL at a density of 2 × 10^6^ cells·mL^−1^. Cells were treated with 50 nm malformin C or DMSO for 4 h then loaded into separate 2 mL chambers and run simultaneously. Experiments were carried out when oxygen concentration in each well was saturated under supra‐atmospheric conditions (~ 400 nm·mL^−1^ O_2_) to ensure the reaction was not rate limited by oxygen. The coupling control protocol (CCP) was followed to measure maximum electron transfer capacity as described previously [28]. Briefly, 1 μm oligomycin, followed by 20 μm FCCP and 1 μm rotenone were added to sequentially to each chamber.

### 2.22 GSH/GSSG measurements

A total of 8500 NCH421k GSCs were seeded into white 96‐well plates in 20 μL HBSS. About 50 nm malformin C or DMSO were added for the indicated times. About 50 μm l‐Buthionine‐sulfoximine (BSO), an inhibitor of glutathione synthesis, was included as a positive control. GSH and GSSG were measured using the GSH‐Glo Glutathione Assay (Promega) according to the manufacturer's protocol.

### 2.23 ROS measurement using roGFP

Lentiviral particles were produced containing the pLVX‐roGFP2 cyto and mito plasmids, which were a gift from Tobias Dick, as described previously [22]. NCH421k cells were transduced at an MOI of 10. After 1 week, 10 000 cells per well were seeded into a PLL and laminin coated black walled 96‐well plate. 24 h later, medium was changed to fluorobrite medium with 2% FBS, and malformin C was applied at the indicated concentrations. About 1 mm H_2_O_2_ was included as a positive control for ROS generation and 2 mm diamide to generate a fully oxidized sample. Fluorescence was measured over 16 h on a PHERAstar FS plate reader (BMG‐Labtech, Ortenberg, Germany). roGFP2 oxidation (OxD_roGFP2_) was calculated as described previously [29].

### 2.24 Staining of protein aggregates

Aggregated proteins were detected using the PROTEOSTAT Aggresome Detection Kit (Enzo Life Sciences, Long Island, NY, USA, ENZ‐51035‐0025). For microscopy, NCH421k GSCs were seeded into laminin/PLL‐coated 12‐well chamber slides (Ibidi) at a density of 40 000 cells in 150 μL. After allowing the cells to adhere overnight, malformin C or MG132 were applied for the times and at the concentrations indicated in the figure. Cells were fixed and permeabilized and according to the manufacturer's protocol, and PROTEOSTAT dye was applied at 1 : 5000 dilution. Images were acquired using a Leica SP5 confocal microscope.

### 2.25 Identification of interacting proteins using mass spectrometry

Azide‐labeled malformin C (Az‐mal. C) was synthesized by Cambridge Peptides. For the pulldown assay, 2 mg protein lysate from NCH421k cells was incubated with 25 μm Az‐mal C or DMSO for 4 h at 4 °C. About 2 mg *N*‐ethylmaleimide (NEM) (Sigma‐Aldrich) was added to block free thiol groups and incubated for 2 h at room temperature. About 20 μL Dibenzocyclooctyne (DBCO) agarose bead slurry (Jena Biosciences, Jena, Germany) was added to each sample and incubated under rotation for 16 h at 4 °C. Beads were washed 4× in 100 mm Tris, 1% SDS, 250 mm NaCl, 5 mm EDTA, pH 8.0, 3× in PBS, 1% SDS and 3× in PBS. Proteins were eluted by incubation with 25 μL 2× LDS loading dye with 2× reducing agent (NU Page, Life Technologies) at 95 °C for 5 min. Proteins (25 μL) were run for 0.5 cm into an SDS/PAGE and the entire piece was cut out and digested using trypsin according to Shevchenko et al. [30], adapted to on a DigestPro MSi robotic system (INTAVIS Bioanalytical Instruments AG, Köln, Germany). A LC–MS/MS analysis was carried out on an Ultimate 3000 UPLC system (Thermo Fisher Scientific, Dreieich, German) directly connected to an Orbitrap Exploris 480 mass spectrometer for a total of 90 min. Peptides were online desalted on a trapping cartridge (Acclaim PepMap 300 C18, 5 μm, 300 Å wide pore; Thermo Fisher Scientific) for 3 min using 30 μL·min^−1^ flow of 0.05% TFA in water. The analytical multistep gradient (300 nL·min^−1^) was performed using a nanoEase MZ Peptide analytical column (300 Å, 1.7 μm, 75 μm × 200 mm, Waters) using solvent A (0.1% formic acid in water) and solvent B (0.1% formic acid in acetonitrile). For 72 min, the concentration of B was linearly ramped from 4% to 30%, followed by a quick ramp to 78%, after 2 min the concentration of B was lowered to 2% and a 10 min equilibration step appended. Eluting peptides were analyzed in the mass spectrometer using data depend acquisition (DDA) mode. A full scan at 120 k resolution (380–1400 m/z, 300% AGC target, 45 ms maxIT) was followed by up to 2 s of MS/MS scans. Peptide features were isolated with a window of 1.4 m/z, fragmented using 26% NCE. Fragment spectra were recorded at 15 k resolution (100% AGC target, 54 ms maxIT). Unassigned and singly charged eluting features were excluded from fragmentation, and dynamic exclusion was set to 35 s. Data analysis was carried out by maxquant (version 1.6.14.0 [31]) using databases extracted from Uniprot.org under default settings (human containing 74 811 entries from 27.02.2020). Identification FDR cutoffs were 0.01 on peptide level and 0.01 on protein level. The match between runs (MBR) option was enabled to transfer peptide identifications across RAW files of the same condition based on accurate retention time and m/z. For quantification, iBAQ values [32] were generated via maxquant.

### 2.26 Click‐iT™ reaction for visualization of Az‐mal. C

NCH421k cells were seeded as described for the staining of protein aggregates. About 50 μm Az‐mal. C was applied for 3–6 h. Alexa fluor 488‐alkyne (Thermo Scientific) was covalently attached to the azide group on the malformin using the Click‐iT™ EdU Imaging kit (Thermo Scientific) according to the manufacturer's protocol with minor modifications. After treatment with Az‐mal C, cells were fixed with 4% paraformaldehyde and permeabilized using 0.1% saponin. After the click reaction, cells were blocked with 5% goat serum and primary antibodies (LAMP2 (Origene, Herford, Germany, H4B4) or TOMM40 (Proteintech, 18409‐1‐AP)) were incubated at a dilution of 1 : 400 at 4 °C overnight. Secondary antibodies (Goat anti‐rabbit Alexa Fluor 647 (A21245, Thermo Scientific) and Goat anti‐mouse Alexa Fluor 555 (A‐21429, Thermo Scientific)) were applied at a dilution of 1 : 500 for 2 h. Cells were mounted using ProLong™ Glass Antifade Mountant with NucBlue™ Stain (Thermo Scientific). Images were acquired using a Leica SP5 confocal microscope.

### 2.27 Analysis of autophagic flux

mCherry‐EGFP‐LC3B was cloned into the pLVX‐puro vector from the pBABE‐puro mCherry‐EGFP‐LC3B construct, which was a gift from Jayanta Debnath (Addgene plasmid #22418) [33] and lentiviral particles were produced as described previously [22]. NCH421k and NCH441 were stably transduced at an MOI of 5 and seeded into laminin/PLL‐coated 8‐well chambers at a density of 45 000 cells per well in 250 μL medium. Malformin C, bafilomycin A1, or tunicamycin were applied as indicated, and nuclei were visualized using Hoechst 33342 (Thermo Scientific) at a concentration of 1 μg·mL^−1^. Images were taken using a Zeiss Cell Observer widefield microscope using the 40× objective. The red and green puncta in the images of the mCherry‐EGFP‐LC3B expressing cells were counted using a macro on imagej [34]. Briefly, was background subtracted from nuclei and puncta images using rolling ball background subtraction, the images were filtered using Gaussian blur and segmented using the ‘find maxima’ tool. The optimal threshold was used for each image separately. Five images were taken per condition and for each image the percentage of cells with 5 or more red or red and green puncta was calculated. Two or three separate experiments for each condition were performed, as indicated in the figure.

### 2.28 Assessment of synergy

NCH421k cells were seeded in 96‐well white plates with 8500 cells per well in 40 μL medium. To determine control curves, cells were treated with 50 μL of a 2× concentration of TMZ or CQ and 10 μL of medium and incubated for 48 h. For drug combinations, cells were treated with 10 μL of a 10× concentration of malformin C and with 50 μL of a 2× concentration of TMZ or CQ. Cell viability was determined after 48 h using the CellTiter‐Glo^®^ assay according to the manufacturer's instructions. Synergy was determined using synergyfinder at https://synergyfinder.org/ [35].

### 2.29 Statistics

Statistical testing was carried out using the functions in Excel 2016 or in graphpad prism, boston, ma, usa 9. Unless otherwise stated, experiments were carried out in biological triplicate and data are represented as the mean ± standard deviation. Unpaired two‐sided Student's *t*‐testing was used to compare means. *P*‐values were represented as follows: * = *P* < 0.05, ** = *P* < 0.01, *** = *P* < 0.001, n.s. = not significant.

## 3 Results

### 3.1 Fungal extract screen reveals vulnerability of GSCs to malformin C

**Fig. 1:**
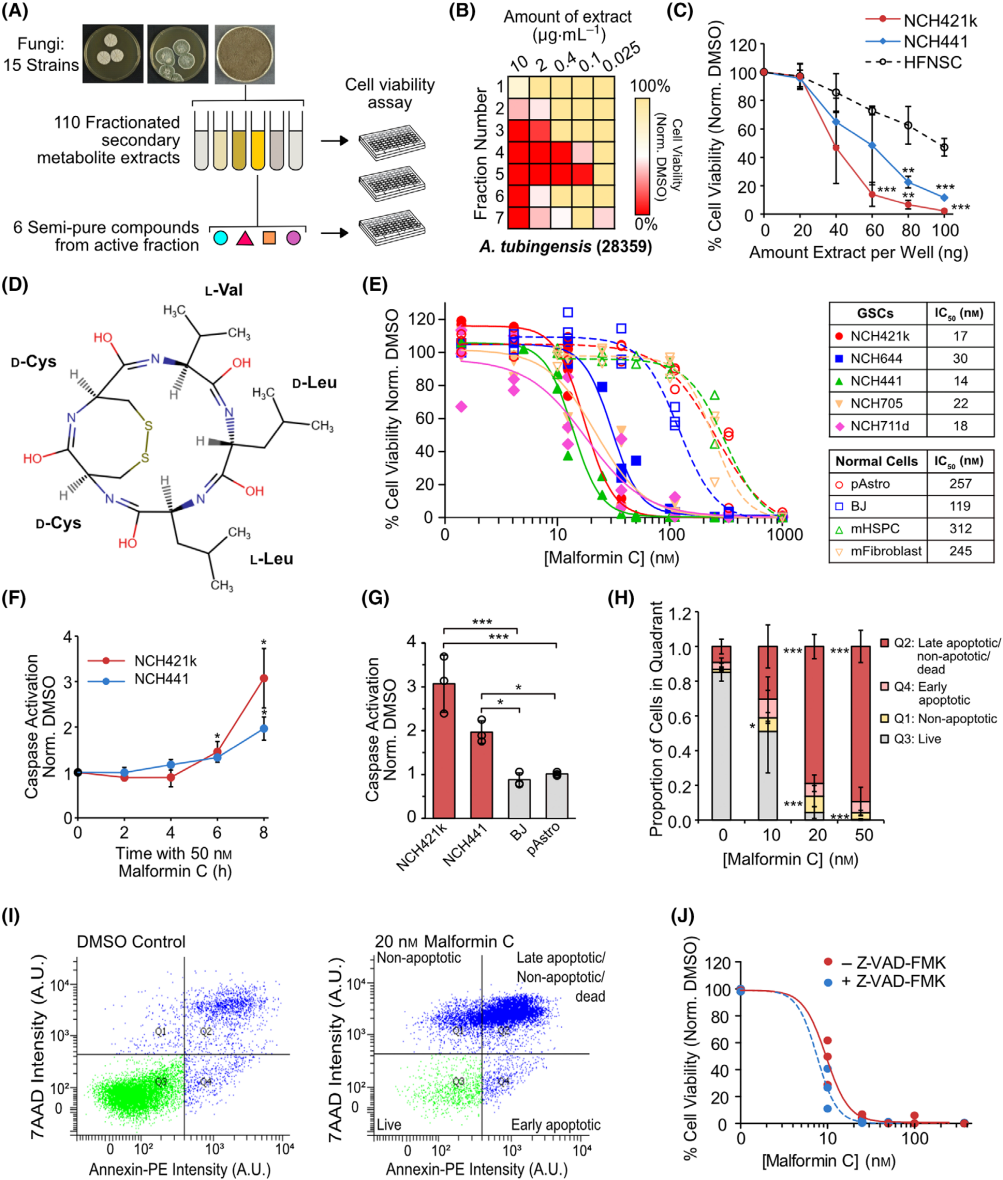
Fungal extract screen reveals vulnerability of glioblastoma stem‐like cells to malformin C. (A) Scheme depicting fungal extract screen workflow. (B) Heatmap showing NCH421k GSC viability after 48 h incubation with different amounts of extract 28 359 fractions. *n* = 1, data representative of two similar experiments. (C) Normalized viability of NCH421k, NCH441 glioblastoma stem‐like cells (GSCs), and human fetal neural stem cells (HFNSC) after 48 h incubation with different amounts of fraction 5 from (B). *n* = 3, mean ± SD, two‐tailed *t*‐test compared to HFNSC, **P < 0.01, ***P < 0.001. (D) Chemical structure of malformin C drawn using the chemical sketch tool from the Protein Data Bank. (E) IC_50_ of malformin C on cell viability of different patient‐derived GSC lines and normal cell lines after 48 h. *n* = 2 or 3, each biological replicate is shown. (F) Caspase activation of NCH421k and NCH441 GSCs over 8 h with 50 nm malformin C. *n* = 3, mean ± SD, two‐tailed *t*‐test compared to DMSO control (=0 h), *P < 0.05. (G) Caspase activation of NCH421k and NCH441 GSCs compared with normal fibroblasts (BJ) and primary astrocytes (pAstro) after 8 h with 50 nm malformin C. *n* = 3, mean ± SD, two‐tailed *t*‐test, *P < 0.05, ***P < 0.001. (H) Annexin V/7AAD staining after 48 h malformin C at different concentrations measured by FACS. Proportion of cells in each quadrant is shown. *n* = 3, mean ± SD, two‐tailed *t*‐test compared with 0 nm (=DMSO), *P < 0.05, ***P < 0.001. (I) Representative image of FACS data from (H). (J) Cell viability of NCH421k GSCs with or without 20 μm pan‐caspase inhibitor Z‐VAD‐FMK upon 48 h incubation with malformin C. *n* = 3, each biological replicate is shown.

In order to identify compounds which could contribute to a therapy against glioblastoma (GB), a natural compound library comprising 110 different marine‐derived fungal extract fractions from 15 strains of *Aspergillus*, *Penicillium*, and *Stilbella* (Table S1) was applied to NCH421k cells, a patient‐derived GSC line, in five different amounts (Fig. 1A). Cell viability was measured after 48 h, revealing 11 fractions to be potent against GSCs, causing a reduction of viability of at least 50% at 400 ng per well (Fig. 1B and Fig. S1). These fractions were tested on a further patient‐derived GSC line, NCH441, and human fetal neural stem cells (HFNSCs), revealing the fractions from *A. tubingensis* IBT 28359 to be less active against the HFNSCs while retaining potent activity against NCH441 (Fig. 1C and Fig. S2A–G). Isolation of pure compounds from these fractions and identification by ultra‐high‐performance liquid chromatography‐diode array detection‐quadrupole time of flight mass spectrometry (UHPLC‐DAD‐QTOF‐MS) suggested that the active compounds belong to the malformins (Fig. 1D and Fig. S2H,I). Being commercially available, malformins C and A_1_ were purchased and tested against different GSC and normal cell lines primary human astrocytes (pAstro), human fibroblasts (BJ), murine bone marrow stromal cells (BMSCs), and murine hematopoietic stem and progenitor cells (HSPCs) (Fig. 1E and Fig. S3A–C). Both malformins were found to be more potent against GSCs than normal cells, with malformin C being more effective than malformin A_1_. Malformin C also demonstrated activity against other cancer cell lines (MDA‐MB‐231, breast; DU145, pancreas; SW480, colon; U87, glioblastoma) but GSCs were the most sensitive of all the cell lines tested (Fig. S3D). Of further note, NCH421k GSCs retained their sensitivity towards malformin C when cultivated in the presence of serum, and U87 remained relatively resistant when serum was removed, suggesting that the sensitivity of the GSCs to malformin is not due stem cell culturing conditions and differentiation state, but rather due to underlying biological properties (Fig. S3E–G). Malformin C was selected over malformin A_1_ for further studies due to its high potency against GSCs.

### 3.2 Malformin C induces apoptotic and nonapoptotic cell death in GSCs

Using a caspase 3/7 activation assay, we found that 50 nm malformin C significantly activated caspases in NCH421k and NCH441 GSCs, but not primary astrocytes (pAstro) or fibroblasts (BJ), within 6–8 h (Fig. 1F,G). Annexin V/7AAD staining of NCH421k GSCs incubated with increasing concentrations of malformin C over 48 h confirmed the induction of apoptotic cell death. However, the presence of cells which were negative for the apoptosis marker annexin V but positive for 7AAD, which can only stain dead cells, also suggested that some cells died by other mechanisms (Fig. 1H,I). The critical involvement of nonapoptotic cell death in the mechanism of action of malformin C in GSCs was confirmed by the fact that pan‐caspase inhibitor Z‐VAD‐FMK did not rescue the malformin C‐induced loss of viability of NCH421k cells (Fig. 1J), although caspases were confirmed to be inactivated (Fig. S3H). Neither was loss of viability rescued by inhibitors of necroptosis (nec‐1), pyroptosis (MCC950), and ferroptosis (fer‐1) (Fig. S3I), leaving the question of the exact cell death pathways induced by malformin C unclear. Interestingly, apoptosis‐inducer staurosporine reduced viability of GSCs and normal cells (astrocytes and fibroblasts) similarly (Fig. S3J), further indicating that malformin C has a mode of action to which GSCs are particularly vulnerable.

### 3.3 Malformin C slows tumor growth *in vivo*

**Fig. 2:**
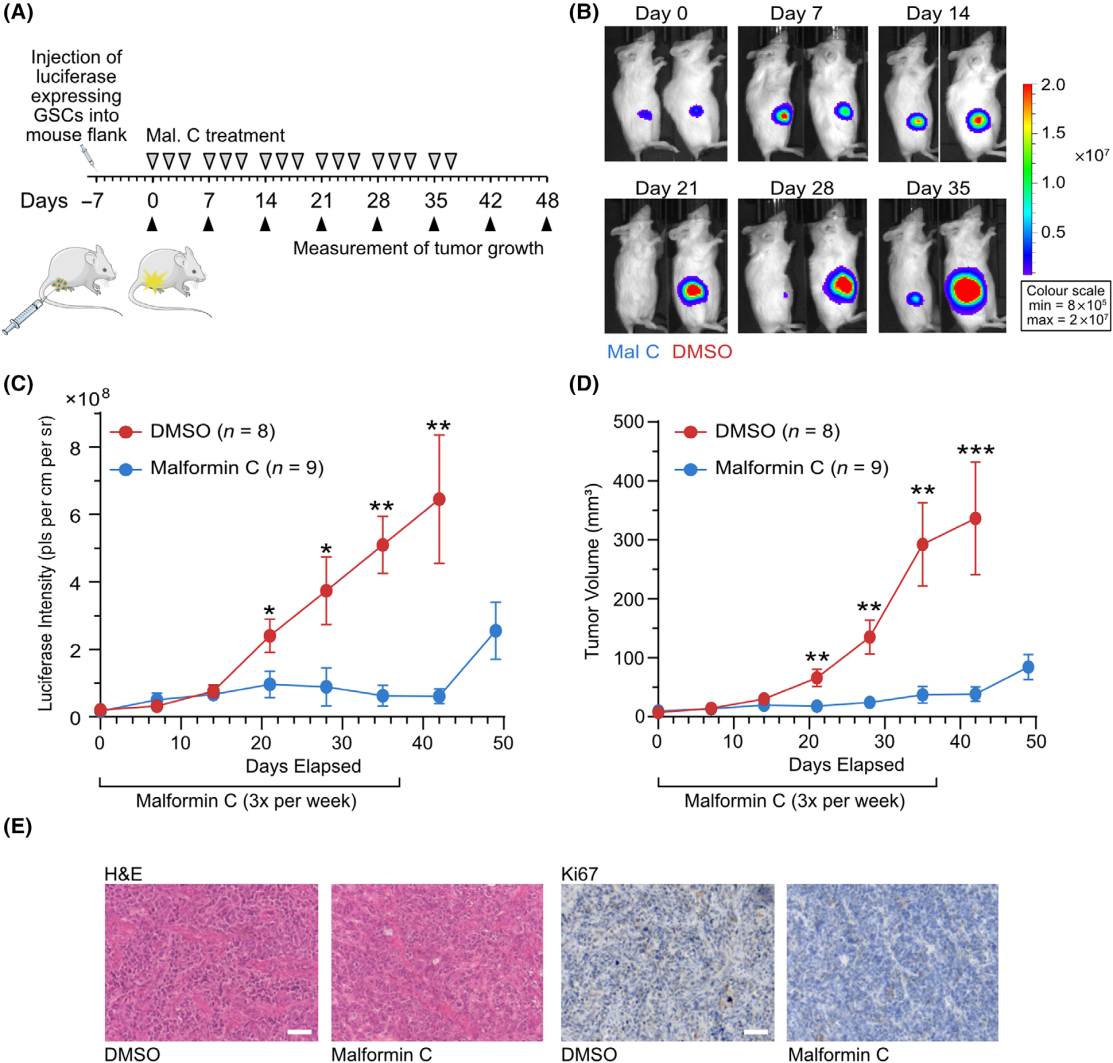
Malformin C slows tumor growth *in vivo*. (A) Scheme showing the workflow of the *in vivo* study (B) Representative bioluminescence imaging of luciferase labeled‐NCH421k cells engrafted into the flanks of NSG mice (DMSO, *n* = 8; mal C = 9 mice). The same two mice are shown at each time point. (C) Luciferase intensity of tumors and (D) tumor volume as determined by caliper measurement in DMSO (red) and malformin C (blue) treated mice. *n* = 8 (DMSO) or 9 (malformin C), mean ± SEM, two‐tailed *t*‐test compared with DMSO, *P < 0.05, **P < 0.01, ***P < 0.001. (E) H&E (left) and Ki67 (right) staining of representative tumors from four DMSO‐treated and four malformin C‐treated mice (scale bar = 100 μm).

The low nanomolar IC_50_ of malformin C against GSCs and the specific induction of cell death in GSCs over normal cells suggests a therapeutic window, warranting *in vivo* evaluation. Malformin C has been previously reported to induce fatal toxicity at doses of over 0.9 mg·kg^−1^ in BDF1 mice, likely due to an acute inflammatory response [20]. We also found that malformin C causes intolerable toxicity to immune‐deficient NOD scid *gamma* (NSG) mice at repeated doses of over 0.9 but 0.5 mg·kg^−1^ administered by *i.p*. injection three times per week was tolerated well. In a pharmacokinetic study, malformin C was found to have a half‐life in the blood plasma of 5.8 h. Although the compound began to accumulate in the brain by 1 h, the equivalent molar concentration was around 1.9 nm, which is lower than the IC_50_ of malformin C observed for GSCs in cell culture (Fig. S4A). Therefore, a flank model was selected to assess the effect of malformin C on glioblastoma growth *in vivo*. To this end, NCH421k GSCs expressing luciferase were injected into the flanks of NSG mice. Seven days later, after engraftment of the cells was confirmed by the appearance of a luminescence signal, mice were treated with malformin C or DMSO (0.5 mg·kg^−1^ 3 × per week) for 37 days. Tumor growth was monitored weekly by luminescence measurement and (for large enough tumors) assessment of volume using calipers for 49 days, or until the tumor reached a maximum volume of 500 mm^3^ (Fig. 2A). Malformin C treatment dramatically suppressed tumor growth and in some cases even led to tumor regression (Fig. 2C,D and Fig. S4B,C). Postmortem hematoxylin and eosin (H&E) staining of the DMSO‐treated tumors displayed the densely packed morphology expected in a glioblastoma and reduced Ki67 expression in malformin C‐treated tumors also reflected slower tumor growth (Fig. 2E). In line with the toxicity study, malformin C treatment did not affect the weights of the mice during the study (Fig. S4D).

### 3.4 Gene expression profiling reveals early oxidative stress, unfolded protein response, and autophagy induction by malformin C in GSCs

**Fig. 3:**
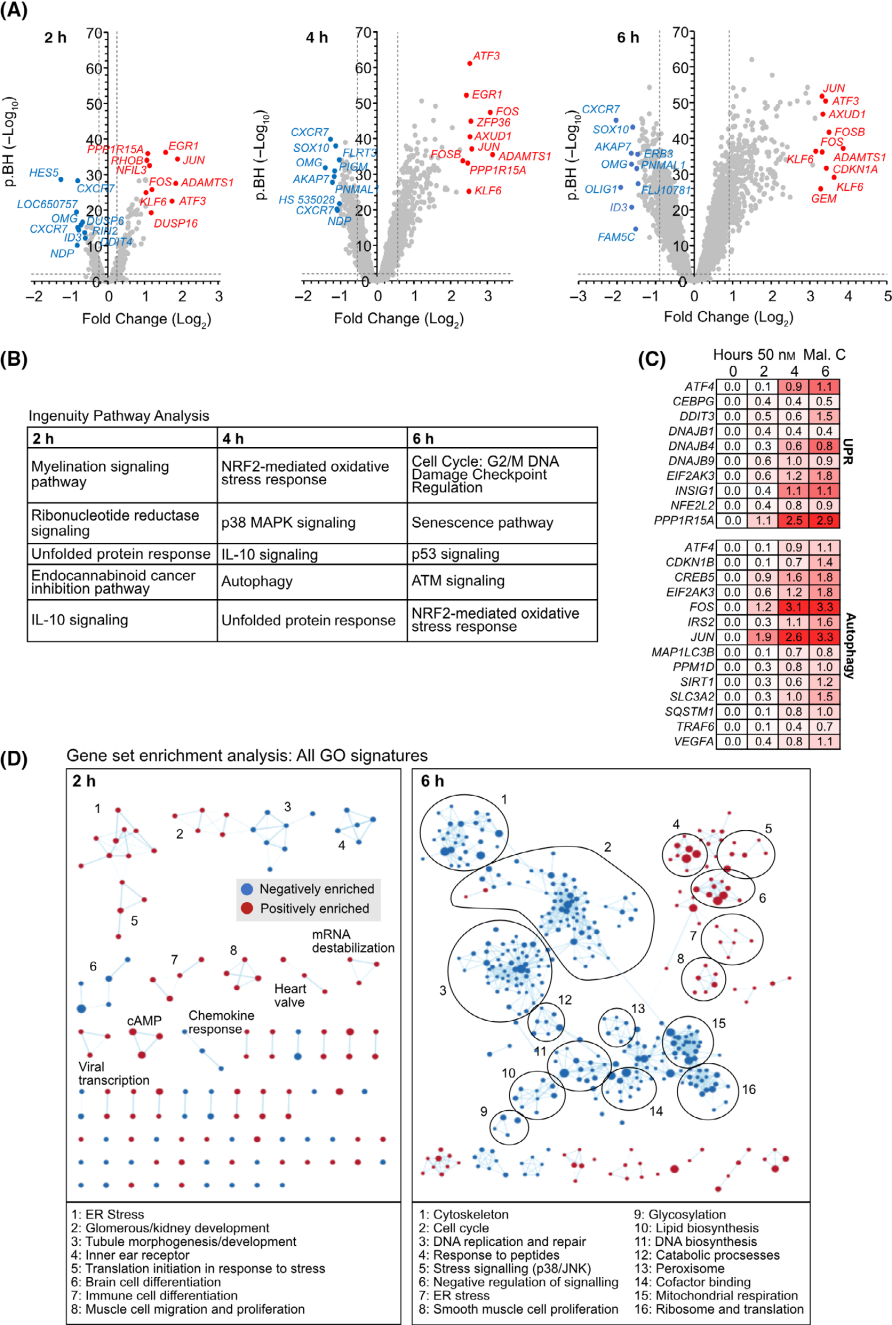
Gene expression profiling reveals early induction of unfolded protein response, oxidative stress, and autophagy by malformin C in glioblastoma stem‐like cells. (A) Volcano plots showing deregulated genes in NCH421k glioblastoma stem‐like cells (GSCs) upon 2, 4, and 6 h incubation with 50 nm malformin C. The top 10 most up‐ and downregulated genes are highlighted in red and blue, respectively. *n* = 3. (B) Table showing the top five deregulated canonical pathways for each time point as determined by Ingenuity pathway analysis. (C) Genes from the Ingenuity ‘unfolded protein response’ and ‘autophagy’ signatures which become deregulated upon malformin C incubation. Log_2_ fold change values compared with DMSO‐treated GSCs are shown. (D) Enrichment map visualization of gene set enrichment analysis of gene expression data using all 6539 annotated gene ontology (GO) signatures in the molecular signatures database at 2 and 6 h.

We next set out to understand the mode of action of malformin C in inducing cell death in GSCs. As we aimed to investigate early gene expression changes before the induction of cell death, NCH421k GSCs were incubated with 50 nm malformin C for 2, 4, and 6 h and RNA was extracted for gene expression profiling. The top deregulated genes for each time point are shown in Fig. 3A. The top 500 deregulated genes (Table S2) were analyzed using Ingenuity Pathway Analysis software, which revealed unfolded protein response (UPR) and oxidative stress to be among the most deregulated pathways at the 2‐h time point, with autophagy and senescence emerging at 4 h, and cell cycle arrest and DNA damage appearing by 6 h (Fig. 3B,C). In a complementary approach, gene set enrichment analysis (GSEA) was carried out using all Gene Ontology signatures. The cytoscape software was used to generate a network of gene set interactions, showing clusters of signatures which share the same genes at the leading edge (Fig. 3D). The top 5 positively and negatively enriched signatures for each time point are listed in Table S3. This also indicated UPR to be an early effect of malformin C, leading to a reduction of translation, proliferation and catabolic processes by 6 h that would be expected when cells are starting to die.

**Fig. 4:**
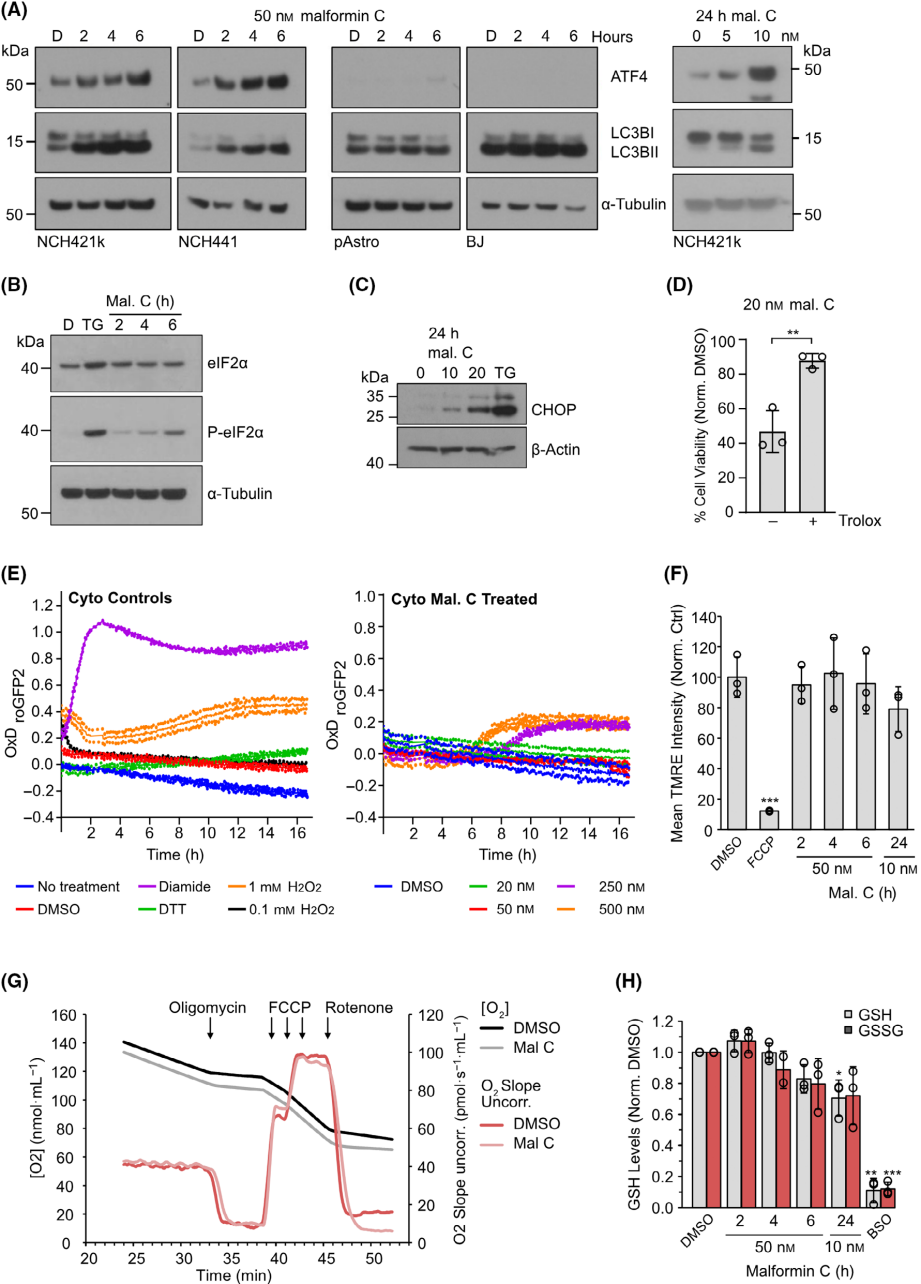
Investigation into the oxidative stress and unfolded protein response induction of malformin C. (A) Western blot showing ATF4 and LC3BI/II protein in (left) glioblastoma stem‐like cells (GSCs) and normal cells (BJ, fibroblasts; pAstro, primary astrocytes) over 6 h incubation with 50 nm malformin C, and (right) NCH421k GSCs after 24 h incubation with lower concentrations of malformin C. α‐tubulin is shown as a loading control, blot representative of three independent experiments. (B) Western blot showing phosphorylated and total levels of EIF2α in NCH421k over 6 h incubation with 50 nm malformin C. (D) DMSO, TG, 300 nm thapsigargin (positive control). α‐tubulin is shown as a loading control, blot representative of two independent experiments. (C) Western blot showing CHOP levels in NCH421k after 24 h incubation with 10 and 20 nm malformin C. TG, 300 nm thapsigargin (positive control). β‐actin is shown as a loading control, blot representative of three independent experiments. (D) Normalized viability of NCH421k GSCs incubated with or without 20 nm malformin C, and with or without 4 h pretreatment with 1 mm Trolox. Data are normalized to the respective DMSO control with or without Trolox (set to 100%, not shown). *n* = 3, mean ± SD, two‐tailed *t*‐test, **P < 0.01. (E) Cytosolic roGFP2 oxidation of NCH421k GSCs with incubation of controls (H_2_O_2_ = positive control for ROS generation; diamide = fully oxidized sample; DTT = reduced sample) or increasing concentrations of malformin C over 16 h. *n* = 1, representative of two similar experiments. (F) Mean TMRE fluorescence intensity, denoting mitochondrial membrane potential, as measured by FACS, in NCH421k GSCs incubated with 50 nm malformin C over 6 h or 10 nm malformin C for 24 h. FCCP (20 μm, 2 h) is a positive control for mitochondrial membrane depolarization. *n* = 3, mean ± SD, two‐tailed *t*‐test, ***P < 0.001, each sample was compared to DMSO. (G) O_2_ consumption during coupling control protocol represented by O_2_ concentration and O_2_ slope in NCH421k GSCs preincubated with 50 nm malformin C for 4 h. *n* = 1, representative of two similar experiments. (H) Glutathione (GSH) and reduced glutathione (GSSG) levels in NCH421k GSCs incubated with 50 nm malformin C over 6 h or 10 nm malformin C for 24 h. *n* = 3, mean ± SD, two‐tailed *t*‐test, *P < 0.05, **P < 0.01, ***P < 0.001, each sample was compared to DMSO. BSO is a positive control for total glutathione depletion.

The gene expression profiling data was validated on the protein level by western blotting. ER stress conditions cause various stress responses, including phosphorylation of PERK, leading to phosphorylation of eIF2α and selective translation of ATF4 protein, phosphorylation of p38, and expression of CHOP. 50 nm malformin C induced phosphorylation of eIF2α and expression of CHOP in NCH421k GSCs and ATF4 expression and p38 phosphorylation in GSCs but not pAstro or BJ cells (Fig. 4A,B and Fig. S5A). Lipidation of LC3BI, which occurs when autophagosomes are formed during autophagy, was similarly induced by malformin C in GSCs (Fig. 4A). Interestingly, γ‐H2AX, which is indicative of DNA damage, was only induced by malformin C after 24 h (Fig. S5B). Further, when cells were pretreated with cycloheximide, which we later show alleviates malformin C‐induced cell death, γ‐H2AX levels were much reduced (Fig. S5C). This supports the observations from the gene expression profiling that malformin C first elicits an ER stress response and autophagy, which likely triggers cell death, later leading to the breakdown of DNA.

### 3.5 Oxidative and proteotoxic stress as key mechanisms in malformin C's toxicity in GSCs

**Fig. 5:**
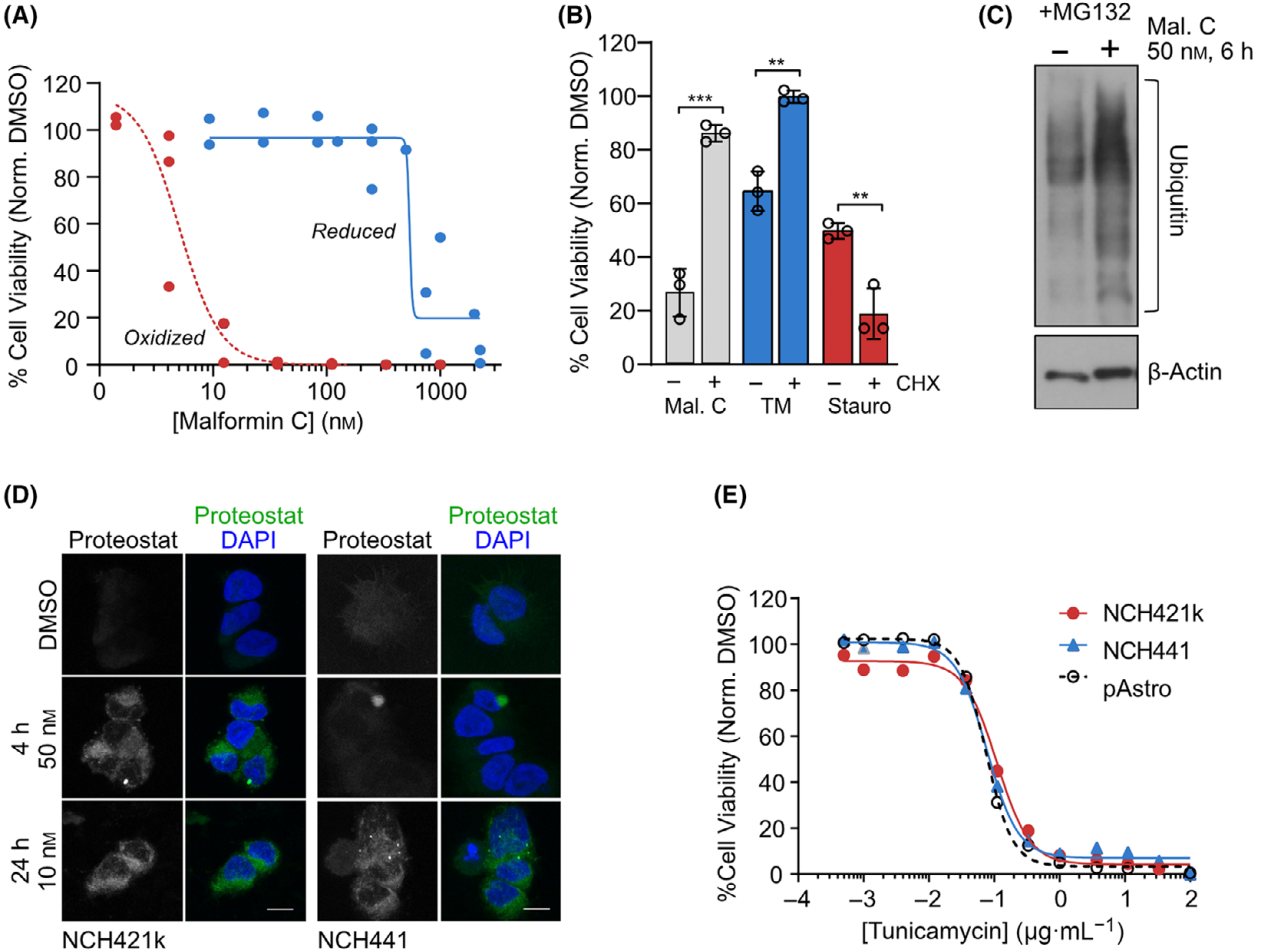
Malformin C disrupts proteostasis by inducing protein aggregation. (A) Cell viability of NCH421k glioblastoma stem‐like cells (GSCs) after 48 h incubation of increasing concentrations of reduced malformin C compared with nonreduced malformin C (*n* = 3). (B) Normalized viability of NCH421k GSCs incubated with or without 20 nm malformin C, 10 μg·mL^−1^ tunicamycin (TM) or 500 nm staurosporine, and with or without 4 h pretreatment with 1 μg·mL^−1^ cycloheximide (CHX). Data are normalized to the respective DMSO control with or without CHX (set to 100%, not shown). *n* = 3, mean ± SD, two‐tailed *t*‐test, **P < 0.01, ***P < 0.001. (C) Western blot of insoluble ubiquitinylated proteins extracted from NCH421k GSCs incubated with 500 nm MG132 for 16 h, followed by 50 nm malformin C for 6 h. β‐actin is shown as a loading control, blot representative of two independent experiments. (D) Proteostat‐stained NCH421k and NCH441 GSCs upon incubation with 50 or 10 nM malformin C for 4 or 24 h, respectively. Images representative from five images from two (NCH421k) or one (NCH441) independent experiment(s). Scale bar = 10 μm. (E) Cell viability of primary astrocytes (pAstro) and NCH421k and NCH441 GSCs after 48 h incubation of increasing concentrations of tunicamycin. *n* = 1, mean of technical duplicates.

In order to confirm the potential oxidative effect of malformin C indicated by the gene expression profiling data, we incubated NCH421k GSCs with the vitamin E analog Trolox, which can protect cells against oxidative stress by scavenging free radicals. Indeed, Trolox significantly reduced GSC sensitivity to malformin C (Fig. 4D). Interestingly, a previous study has implicated ER stress and autophagy, along with mitochondrial disruption and generation of reactive oxygen species (ROS), in malformin A_1_‐induced cell death in prostate cancer cells. As induction of ROS was the earliest phenotype observed in that study, the authors postulated that the reactive thiol groups of malformin A_1_ might deplete the cellular pool of glutathione (GSH), leading to an accumulation of ROS and subsequent ER stress, autophagy, mitochondrial dysfunction and DNA damage, culminating in cell death [21]. Therefore, we sought to determine whether malformin C causes similar effects in GSCs. Intracellular ROS levels were measured using NCH421k GSCs stably expressing redox sensitive cytoplasmic and mitochondrial GFP probes. Malformin C was applied at concentrations ranging from 20 to 500 nm and the wavelength of GFP fluorescence monitored over 16 h. Although malformin C induced cytoplasmic ROS at high concentrations, there was no observation of ROS induction at lower malformin concentrations and early time points (Fig. 4E and Fig. S5D). Mitochondrial function was assessed by measuring mitochondrial membrane potential using tetramethylrhodamine ethyl ester (TMRE) staining and quantification by FACS and following oxygen consumption as an indicator of rate of oxidative phosphorylation using an oxygraph. Malformin did not significantly reduce the mitochondrial membrane potential of NCH421k GSCs (Fig. 4F). Nor were any changes in oxygen consumption observed when NCH421k cells were incubated with 50 nm malformin C for 4 h compared with DMSO (Fig. 4G). Finally, GSH and GSSG (reduced glutathione) levels were measured in NCH421k GSCs using a GSH/GSSG Glo assay. While malformin C does seem to reduce GSH levels over 24 h, GSSG levels also drop, suggesting an inhibition of glutathione synthesis rather than reduction (Fig. 4H). However, when malformin C's disulfide bond was disrupted by incubation with reducing agent DTT, activity against NCH421k GSCs was severely diminished (IC_50_ > 500 nm) (Fig. 5A). Despite the protective effect of Trolox, taken together, these experiments suggest that in the context of GSCs, the dramatic cell death induced by malformin C is not caused by glutathione depletion and subsequent ROS generation, although the reactive thiol groups do appear to have a function in the mode of action of the compound. Given the importance of these groups, we hypothesized that malformin C could cause proteotoxic stress in GSCs by reacting with exposed thiols of many proteins which could lead to protein aggregation, inducing UPR and oxidative stress, autophagy and cell death. Supporting this, incubating GSCs with translation inhibitor cycloheximide (CHX) as a means of alleviating proteotoxic stress reduced the sensitivity of GSCs to malformin C. Toxicity effects of the ER stress inducer tunicamycin (TM) were also rescued by CHX, whereas toxicity of staurosporine was not (Fig. 5B). Supporting this, malformin C caused accumulation of insoluble ubiquitinylated proteins in NCH421k GSCs when the proteasome was blocked using MG132 (Fig. 5C), and protein aggregates were visualized in GSCs by staining with Proteostat, a rotor dye which only fluoresces when bound to denatured proteins (Fig. 5D). Interestingly, other compounds containing disulfide bonds did not selectively reduce viability in GSCs compared with astrocytes (Fig. S6), and GSCs were not more sensitive than astrocytes to tunicamycin (Fig. 5E). Taken together, this suggests that the proteotoxic effect of malformin C is likely not solely responsible for the vulnerability of GSCs to this compound.

### 3.6 Click‐chemistry based target identification reveals association of malformin C to membrane transport and lysosomal proteins

**Fig. 6:**
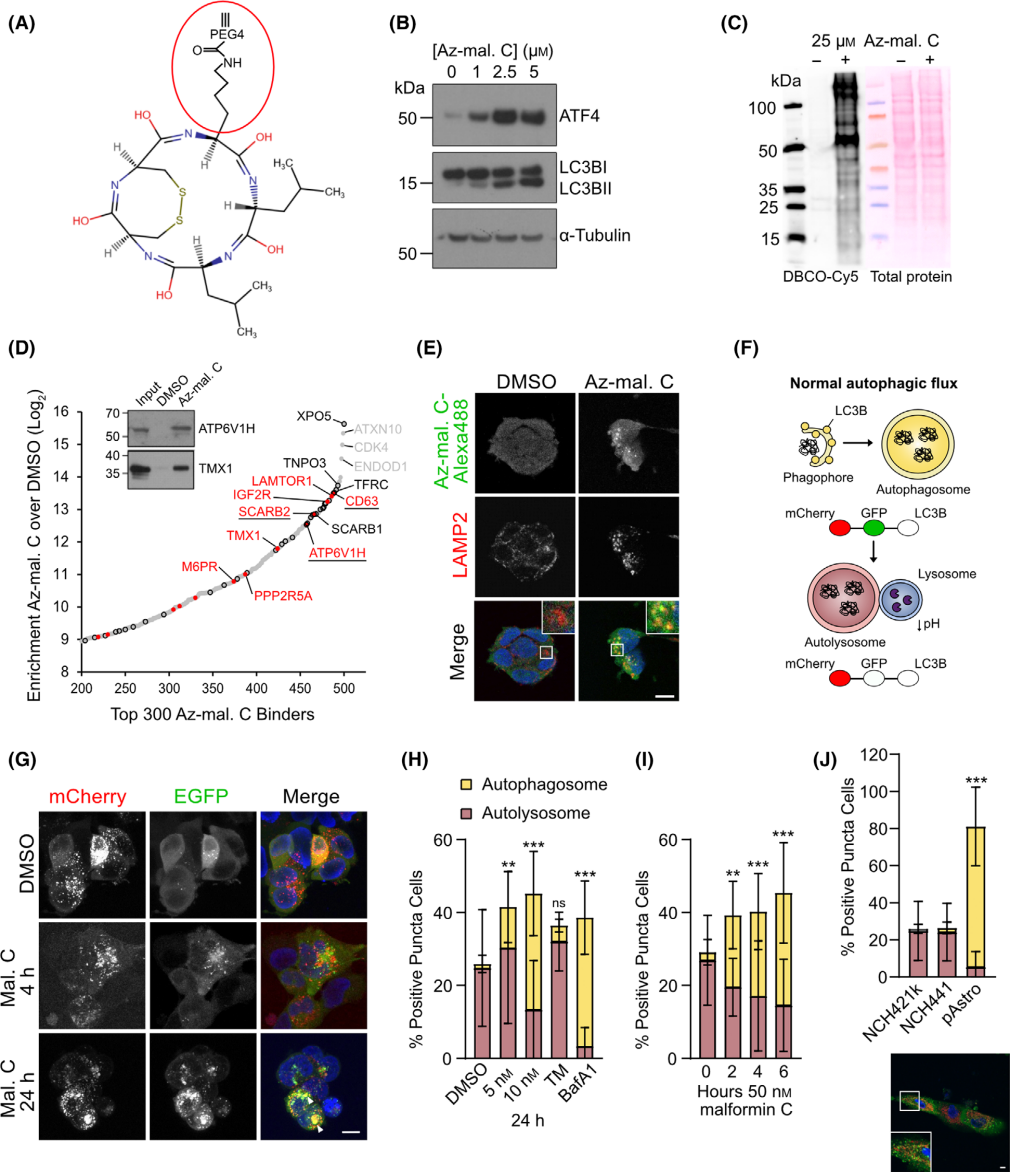
Click‐chemistry based target identification reveals lysosomotropic properties of malformin C, leading to disruption of autophagic flux. (A) Structure of azide‐modified malformin C (Az‐mal. C). (B) Western blot showing ATF4 and LC3BI/II protein levels in NCH421k glioblastoma stem‐like cells (GSCs) after 24 h incubation with increasing concentrations of Az‐mal. C. α‐tubulin is shown as a loading control, blot representative of two independent experiments. (C) Separated and transferred DBCO‐Cy5‐labeled proteins (left) or total protein (right, ponceau staining) in total NCH421k lysate after incubation with 25 μm Az‐mal. C or DMSO for 4 h. Representative of four independent replicates. (D) Rank plot of the 300 most highly abundant proteins identified in Az‐mal. C pull down by mass spectrometry. Proteins marked in red are part of the CLEAR (coordinated lysosomal expression and regulation) signaling pathway; proteins circled in black are membrane transport proteins. Red proteins circled/underlined in black are involved in both. Inset: Confirmation of enrichment of ATP6V1H and TMX1 on Az‐mal. C bound beads by western blot, blot representative of a single experiment, which was independent of the pull‐downs carried out for the mass spectrometry analysis. (E) Confocal microscopy images of DMSO or Az‐mal. C with covalently bound Alexa fluor 488‐alkyne (green), along with immunostaining of lysosomal marker LAMP2 (red), in NCH421k GSCs. Cells were incubated with 30 μm Az‐mal. C for 6 h with subsequent binding of Alexa fluor 488. Representative image from one of 10 gathered over two independent experiments. Scale bar = 10 μm. (F) Scheme explaining autophagic flux measurement using the mCherry‐GFP‐LC3B fusion protein. (G) Representative images from three experiments of NCH421k GSCs stably expressing mCherry‐GFP‐LC3B after incubation with 50 or 10 nm malformin C for 4 or 24 h, respectively. (H) Quantification of % positive NCH421k GSCs for autolysosomes (red) or autophagosomes (yellow) after 24 h incubation with 5 or 10 nm malformin C, 1 μg·mL^−1^ tunicamycin (TM), or 30 nm bafilomycin A1. Data are representative of 3 (DMSO, malformin C) or 2 (TM, Baf A1) independent experiments with five images taken for each experiment, mean ± SD, one‐way ANOVA, **P < 0.01, ***P < 0.001, ns, not significant (numbers of autophagosomes compared with DMSO). (I) As (H) but NCH421k GSCs were incubated with 50 nm malformin C over 6 h. (J) Quantification of autophagic flux in GSCs compared with primary astrocytes (10 images from two experiments) (mean ± SD, one‐way ANOVA, ***P < 0.001), with a representative image of pAstro stably expressing mCherry‐GFP‐LC3B. Scale bar = 10 μm.

In a bid to further understand the mechanism by which malformin C exerts its dramatic effects in GSCs, we used a clickable azide‐labeled version of malformin C (named ‘Az‐mal. C’) (Fig. 6A) to pull down and identify directly interacting proteins and explore the intracellular distribution of the compound. Although the modification reduced the activity of malformin C, Az‐mal. C still induced ATF4 expression and LC3BI lipidation in GSCs, culminating in cell death which was stronger in GSCs compared with astrocytes (Fig. 6B and Fig. S7A). We therefore assumed that Az‐mal. C has a similar effect to malformin C on GSCs, but that higher amounts of the compound are required. For identifying interacting proteins, Az‐mal. C or DMSO was applied to GSC lysate, followed by addition of DBCO‐Cy5 for visualization, or DBCO‐agarose beads for the pull‐down and mass spectrometric identification of Az‐mal. C‐bound proteins. Many fluorescent bands were revealed in the DBCO‐Cy5 stained lysates incubated with Az‐mal. C, suggesting interaction with many proteins (Fig. 6C). This was confirmed in the mass spectrometry analysis, as peptides corresponding to around 500 different proteins were found to be enriched in the Az‐mal. C lysate over the DMSO control (Fig. 6D). The top 20 enriched proteins are listed in Table S4, many of which are upregulated in glioblastoma (11/20). Some of the identified proteins (TOMM40, SQLE, TMX1, TFRC, ATPV61H, PRDX6) were confirmed by repeating the pull‐down and performing western blot analysis on the eluates (Fig. 6D and Fig. S7B). It was striking to see a high number of membrane transport and lysosomal proteins enriched, with pathway analysis of the top 300 enriched candidates revealing proteins belonging to the coordinated lysosomal expression and regulation (CLEAR) signaling pathway, and involved in molecular transport and protein trafficking to be highly represented (Tables S5 and S6). Of further interest was the enrichment of several proteins involved in cholesterol biosynthesis. Confocal microscopy of NCH421k cells incubated with 50 μm Az‐mal. C for 3 or 6 h, followed by labeling with alkyne‐Alexa 488 revealed a plasma membrane, cytosolic, and punctuate distribution of Az‐mal C. Staining of lysosomes and mitochondria showed that the punctuate staining was localized in lysosomes rather than mitochondria (Fig. 6E and Fig. S7C).

### 3.7 Malformin C blocks autophagic flux

In light of the implication of malformin C in autophagy established in our gene expression profiling and LC3B protein analyses, the enrichment of lysosomal proteins bound to Az‐mal. C seemed highly relevant and inspired us to investigate the effect of malformin C on autophagy in more detail. In order to accurately monitor autophagic flux, we generated NCH421k and NCH441 GSCs stably expressing an mCherry‐GFP‐LC3B fusion protein. When autophagy is induced, the fusion protein becomes localized to autophagosomes forming red and green puncta (which appear yellow), visible by microscopy. When the autolysosome is formed, the acidic environment quenches GFP fluorescence, resulting in puncta appearing red only. If autophagic flux is blocked, the puncta retain their GFP fluorescence (Fig. 6F). As indicated by an accumulation of autophagosomes already at 2 h, malformin C disrupted autophagic flux in GSCs (Fig. 6G–I and Fig. S7D–F). vATPase inhibitor bafilomycin A1 was used as a positive control as an autophagy inhibitor which prevents acidification of the autolysosome. As a negative control, tunicamycin did not significantly affect autophagosome accumulation. The appearance of enlarged yellow puncta (indicated by white triangles) suggests that autolysosomes were formed but that the lysosomal function was impaired by malformin C. Furthermore, the very low levels of autophagosomes compared with autolysosomes in GSCs were striking, suggesting high basal autophagic flux in these cells. For comparison, primary astrocytes transduced with the mCherry‐GFP‐LC3B fusion protein exhibited many more autophagosomes, suggesting that autophagy could be differentially regulated in GSCs and normal cells (Fig. 6J). We next assessed whether the block in autophagic flux might be caused by induction of lysosomal membrane permeabilization (LMP) by malformin C, using the cysteine protease inhibitor K777. While K777 rescued the loss of cell viability caused by the well‐known LMP inducer LLME, malformin C‐induced cell death was not alleviated, leading us to conclude that malformin C does not directly affect lysosomal membrane integrity (Fig. S7F).

### 3.8 Malformin C acts synergistically with temozolomide and chloroquine in GSCs

**Fig. 7:**
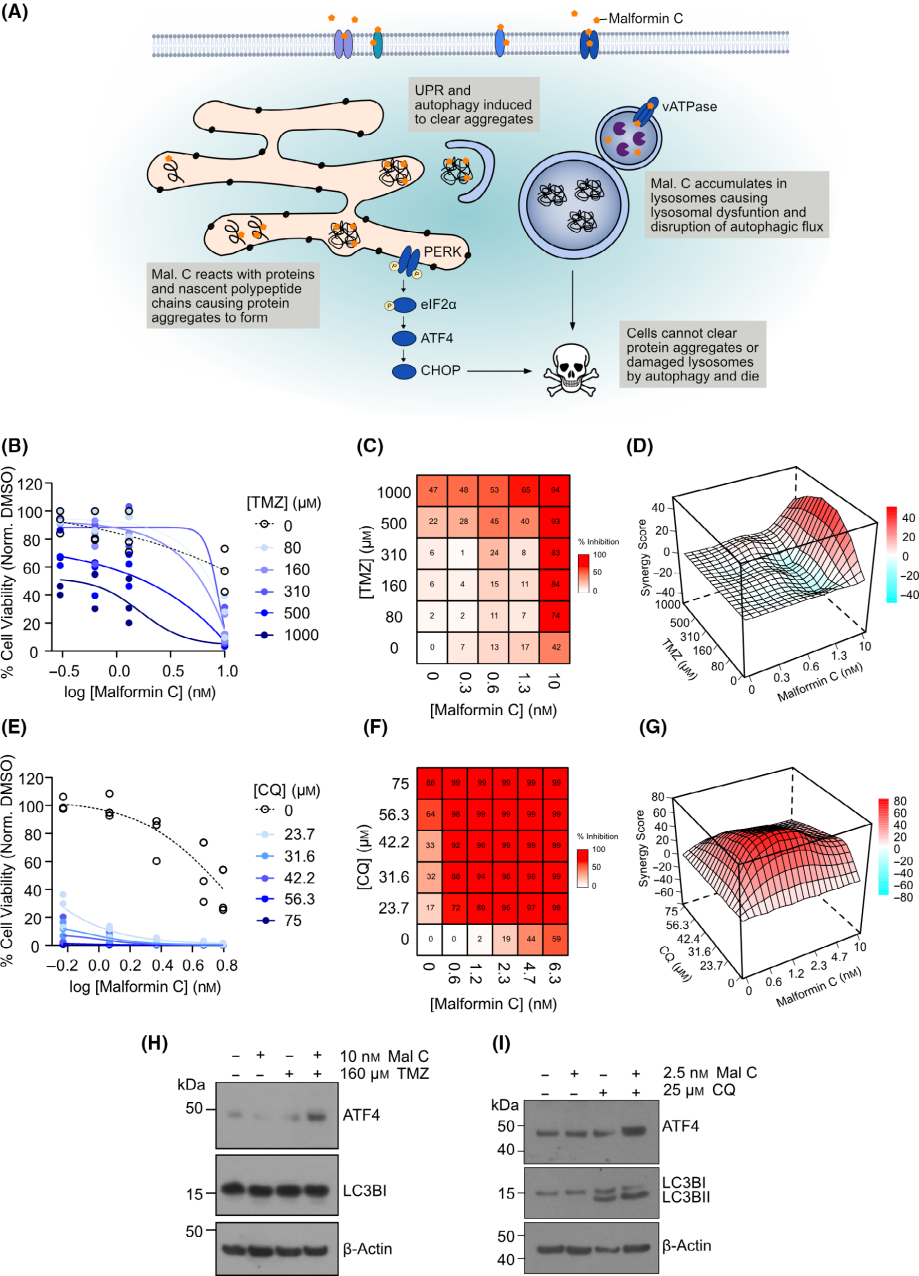
Malformin C acts synergistically with temozolomide and chloroquine in glioblastoma stem‐like cells. (A) Scheme depicting the mechanism of action of malformin C in glioblastoma stem‐like cells (GSCs). UPR: Unfolded protein response. (B) Normalized cell viability of NCH421k GSCs with increasing concentrations of malformin and temozolomide (TMZ). *n* = 3. (C) Heatmap showing % inhibition of NCH421k with combination treatment of malformin C and TMZ at different concentrations. *n* = 3. (D) 3D contour plot showing the ZIP synergy score of the combinations. *n* = 3. (E) Normalized cell viability of NCH421k GSCs with increasing concentrations of malformin C and chloroquine (CQ). *n* = 3. (F) Heatmap showing % inhibition of NCH421k with combination treatment of malformin C and CQ at different concentrations. *n* = 3. (G) 3D contour plot showing the ZIP synergy score of the combinations. *n* = 3. (H, I) Western blots showing ATF4 and LC3BI/II levels in NCH421k GSCs with single treatments of malformin C, TMZ and CQ, or combination treatment at the same concentrations. β‐actin is shown as a loading control, blot representative of two independent experiments.

Our data suggest that malformin C has multiple effects in GSCs, eliciting a strong unfolded protein response by induction of protein aggregation, while simultaneously preventing the autophagic clearance of aggregates, driving the cells to death (Fig. 7A). While dramatically impairing tumor growth *in vivo*, when the treatment was stopped after 37 days, growth resumed, suggesting that some cells within the tumor may be resistant to malformin C. Therefore, in order to further potentiate the effects of the compound, we tested whether malformin C could act synergistically with DNA alkylating agent temozolomide (TMZ), which is currently used in the standard therapeutic regimen for GB, and autophagy inhibitor chloroquine (CQ). Although a synergistic effect could be observed for both compounds, the combination with CQ was much stronger (Fig. 7B–G). Combination of malformin C and TMZ led to increased ATF4 expression but no enhanced lipidation of LC3B, whereas combination with CQ increased both ATF4 expression and LC3B lipidation (Fig. 7H,I). This further supports the role of the lysosomes and autophagy in the mechanism of action of malformin C and warrants the study of malformin C in combination with autophagy inhibitors further.

## 4 Discussion

Glioblastoma is an extremely aggressive brain tumor with limited treatment options. Here we sought to identify candidate antitumor molecules that eradicate patient‐derived glioblastoma stem‐like cells using a fungal extract screen. Due to its high potency against GSCs derived from different patients compared with normal cells, malformin C captured our attention as a putative therapeutic compound.

Many biological activities have been attributed to the malformins, including anticancer activity [13, 16, 17, 18, 19, 20, 21]. In a recent study, malformin C was evaluated *in vivo* as a therapeutic agent against colon cancer. Although the authors showed a tumor slowing effect, the authors concluded that the therapeutic index of malformin C is too low to be an anticancer drug [20]. In our study, we found that GSCs are more sensitive (by around 10‐fold) to malformin C than the cell lines investigated by Wang et al., and the tumor inhibitory effect of malformin C on GSCs *in vivo* was highly significant, which suggests that the compound could still be an attractive option as a therapy for glioblastoma. That said, the toxicity of malformin C does indeed limit the dose which can be administered to patients, which is particularly relevant for ensuring accumulation in the brain. We found that combining malformin with temozolomide or chloroquine potentiated the effects of the drug against GSCs *in vitro*. More work will be required to investigate the efficacy of these combinations *in vivo*, and it will be particularly interesting to determine whether combining malformin C with chloroquine or other inhibitors of autophagy can elicit tumor inhibitory effects in an orthotopic model of glioblastoma. In addition, as we found that malformin C also induces cell death in other cancer cell lines *in vitro*, malformin C could be useful for the treatment of other aggressive cancers, such as breast or prostate, which would be independent of the ability of the compound to penetrate the brain.

Our study also sheds much light on the mode of action of malformin C in GSCs. We found that malformin C causes dramatic cell death via apoptotic and nonapoptotic pathways. Gene expression profiling suggested that ER stress and autophagy were among the earliest events induced by the compound. Although malformin A_1_ has been suggested to induce reactive oxygen species and mitochondrial damage in prostate cancer cells at early time points, perhaps by impairing glutathione via its redox active disulfide group [21], this did not appear to be the case in GSCs. Rather, using chemical proteomics, we found that malformin C binds to many proteins, including multiple membrane transporters and proteins involved in cholesterol biosynthesis, and accumulates in lysosomes. We also showed that the compound causes proteotoxic stress by inducing protein aggregation, while also blocking autophagic flux. On the other hand, our data do not rule out the possibility that the dramatic effects of malformin C in GSCs could be enhanced by inhibition of specific proteins which are particularly relevant to GB biology; for example, the further investigation of the effects of malformin C on cholesterol in GSCs could offer deeper insight into the mode of action of the compound.

The binding of Az‐mal. C to numerous membrane transporters might reveal some insight into the way malformin C is taken up by cells. Of particular note, two of the highly enriched membrane proteins, transferrin receptor protein 1 (TFRC) and scavenger receptor class B member 1 (SCARB1), have been proposed as targets for thiol‐mediated uptake [36, 37]. The binding of Az‐mal. C to these and other membrane transporters suggest that malformin C might react with the cysteines in membrane proteins and use them to enter the cells. Once inside, malformin C likely reacts with exposed thiols on many proteins, leading to protein aggregation and ER stress. As covalent inhibitors targeting cysteines are of interest in efforts to target traditionally ‘undruggable’ proteins [38], the covalent chemistry involved in the mode of action of malformin C renders it particularly interesting as a lead compound.

Sustained ER stress can culminate in cell death by apoptosis via activation of the PERK signaling branch of the unfolded protein response [39], which may account for the apoptosis observed in malformin C‐treated GSCs. On the other hand, lysosomal damage can induce cell death via other modalities, such as necrosis and necroptosis [40]. Attempts to delineate the precise cell death modalities triggered by malformin C using various inhibitors were unsuccessful. This could be explained by even further noncanonical cell death modalities that may be triggered by malformin C, or perhaps more likely by the possibility of the compound triggering multiple cell death pathways simultaneously. Considering that amines with hydrophobic side chains are classed as lysosomotropic detergents [41], it was not entirely surprising that we found malformin C, with its three hydrophobic amino acids incorporated in the pentapeptide ring, to accumulate in the lysosomes and impact autophagic flux. Further, the binding of malformin C to membrane proteins that are subjected to receptor‐mediated endocytosis, such as the TFRC [42], could provide a further route for the transport of the compound to the lysosomes. Taken together, it seems that the proteotoxic and lysosomotropic effects of malformin act in concert to bring about catastrophic cell death in GSCs.

Mounting evidence suggests that autophagy is critical for glioma initiation and growth [43] and is exploited by many different tumor types, including glioblastoma, for maintaining cellular fitness in the face of metabolic and genotoxic stress [44]. Accordingly, it seems that glioblastoma could be particularly susceptible to intervention along the autophagy and lysosomal pathways [45, 46, 47]. Indeed, the autophagy inhibitor chloroquine, which we found to act highly synergistically with malformin C in GSCs, has been tested in clinical trials for GB and is associated with improved remission and prolonged patient survival [48].

Interestingly, we observed drastically low numbers of autolysosomes in GSCs compared with lysosomes, eluding to higher autophagic flux in GSCs, which could suggest a dependency of GSCs on autophagy. Along these lines, a recent study has shown that hematopoietic stem cells (HSCs) preferentially traffic protein aggregates to aggrosomes rather than the proteasome and that HSCs are dependent on high levels of selective autophagy to clear these aggregates [49]. It has also been reported that neural stem cells are dependent on lysosome activation to clear aggregates which form during aging [50], further suggesting that cells with stem‐like characteristics, such as the GSCs used in this study, are highly dependent on a tight regulation of proteostasis via autophagic clearance.

## 5 Conclusions

In conclusion, using a fungal extract screening approach, this study revealed malformin C to be potent against glioblastoma; providing mechanistic insights into the mode of action of the compound which highlight the relevance of proteostasis and autophagy as a therapeutic vulnerability in this deadly disease.

## Conflict of interest

The authors declare no conflict of interest.

## Author contributions

VG and TOL conceived the project. CH‐M provided the glioblastoma stem‐like cells and critically reviewed the work for intellectual content. EP, SE, SS, MG, VJ, FB, and GM performed most experiments with the following exceptions: SK produced the fungal extract library and identified the malformins; DP‐F performed oxidation experiments; DH and MS performed proteomics analysis. EP wrote the manuscript with feedback from all authors.

### Peer review

The peer review history for this article is available at https://www.webofscience.com/api/gateway/wos/peer‐review/10.1002/1878‐0261.13756.

## Supporting information


**Fig. S1.** Results of the screen.
**Fig. S2.** Active fractions tested in further cell lines and identification of malformin C.
**Fig. S3.** Characterization of cell death induced by the malformins.
**Fig. S4.** Malformin C slows tumor growth *in vivo*.
**Fig. S5.** Investigation of malformin C‐induced DNA damage and oxidative stress in glioblastoma stem‐like cells.
**Fig. S6.** Glioblastoma stem‐like cells are not sensitive to other compounds containing disulfide bonds.
**Fig. S7.** Malformin C disrupts autophagic flux in glioblastoma stem‐like cells.

**Table S1.** Fungal strains used in the extract screen and their sources.
**Table S2.** The top 500 deregulated genes after incubation of 50 nm malformin C after 2, 4 and 6 h.
**Table S3.** Top positively and negatively enriched signatures from gene set enrichment profiling using the gene ontology collection.
**Table S4.** Top 20 most enriched proteins identified in Az‐mal. C pulldown.
**Table S5.** Top 10 ‘canonical pathways’ identified from the 300 most enriched proteins from the Az‐mal. C pulldown.
**Table S6.** Top 5 ‘molecular functions’ identified from the 300 most enriched proteins from the Az‐mal. C pulldown.

## Data Availability

The gene expression profiling data have been submitted to the Gene Expression Omnibus under accession GSE231595. The mass spectrometry proteomics data have been deposited at the ProteomeXchange Consortium via the PRIDE [51] partner repository with the dataset identifier PXD046258. Other data that support the findings of this study are available from the corresponding author upon request.
