# Supplementary material for: Malformin C preferentially kills glioblastoma stem‐like cells via concerted induction of proteotoxic stress and autophagic flux blockade
Source: Mol Oncol. 2024 Oct 27;19(3):785–807. doi: 10.1002/1878-0261.13756 (PMC11887673; doi:10.1002/1878-0261.13756)
Supplement: Supplementary file 1 — Fig. S1. Results of the screen. Fig. S2. Active fractions tested in further cell lines and identification of malformin C. Fig. S3. Characterization of cell death induced by the malformins. Fig. S4. Malformin C slows tumor growth in vivo. Fig. S5. Investigation of malformin C‐induced DNA damage and oxidative stress in glioblastoma stem‐like cells. Fig. S6. Glioblastoma stem‐like cells are not sensitive to other compounds containing disulfide bonds. Fig. S7. Malformin C disrupts autophagic flux in glioblastoma stem‐like cells. [file MOL2-19-785-s002.pdf]

Malformin C preferentially kills glioblastoma stem-like cells via concerted induction of proteotoxic stress and autophagic flux blockade

Emma Phillips[1], Sizèd van Enk[1], Sara Kildgaard[2], Silja Schlue[1], Mona Göttmann[1], Victoria Jennings[1], Frederic Bethke[1], Gabriele Müller[1], Christel Herold-Mende[3], Daniel Pastor-Flores[4], Martin Schneider[5], Dominic Helm[5], Thomas Ostenfeld Larsen[2], Violaine Goidts[1]

Supplementary Figures

Supplementary Figure S1

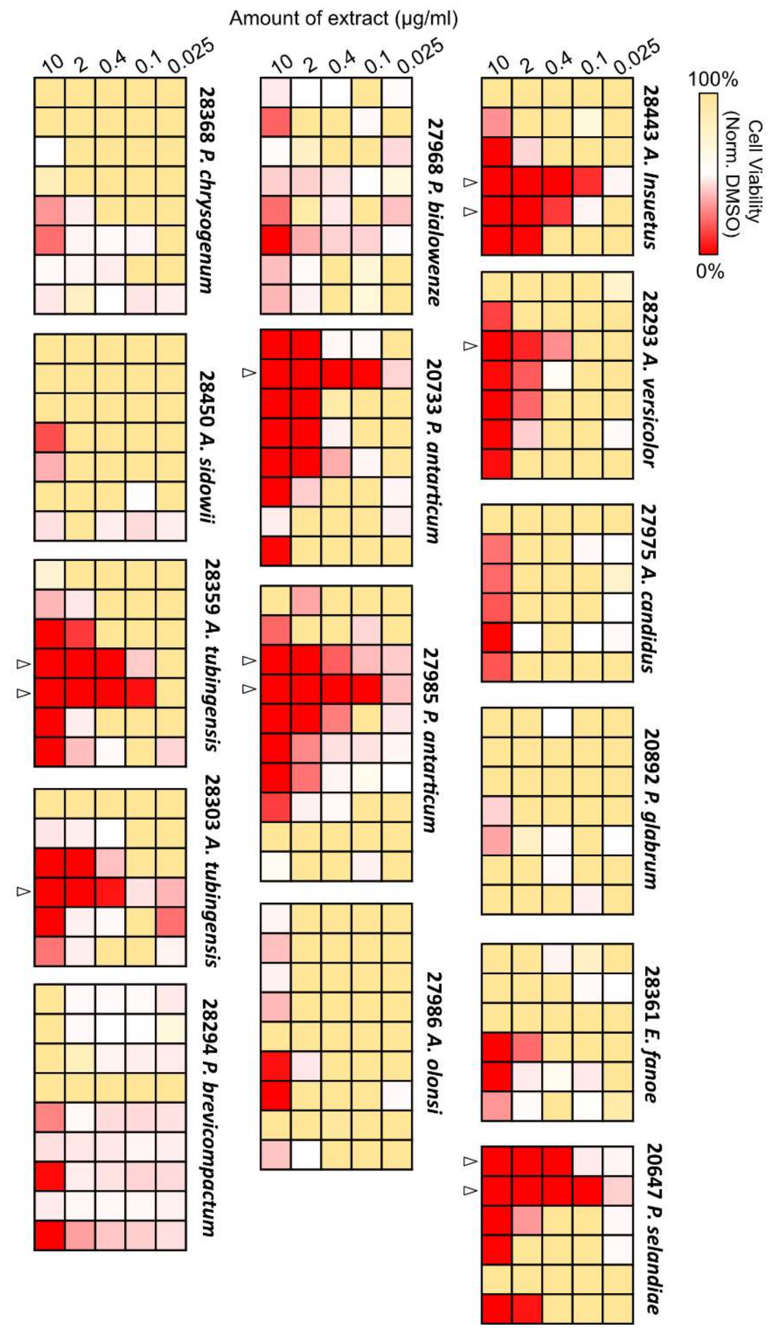

Results of the screen

Heatmap showing NCH421k glioblastoma stem-like cell viability after 48 hours incubation with different amounts of each extract, n=1. Triangles indicate fractions which were considered “active” and investigated in further cell lines.

Supplementary Figure S2

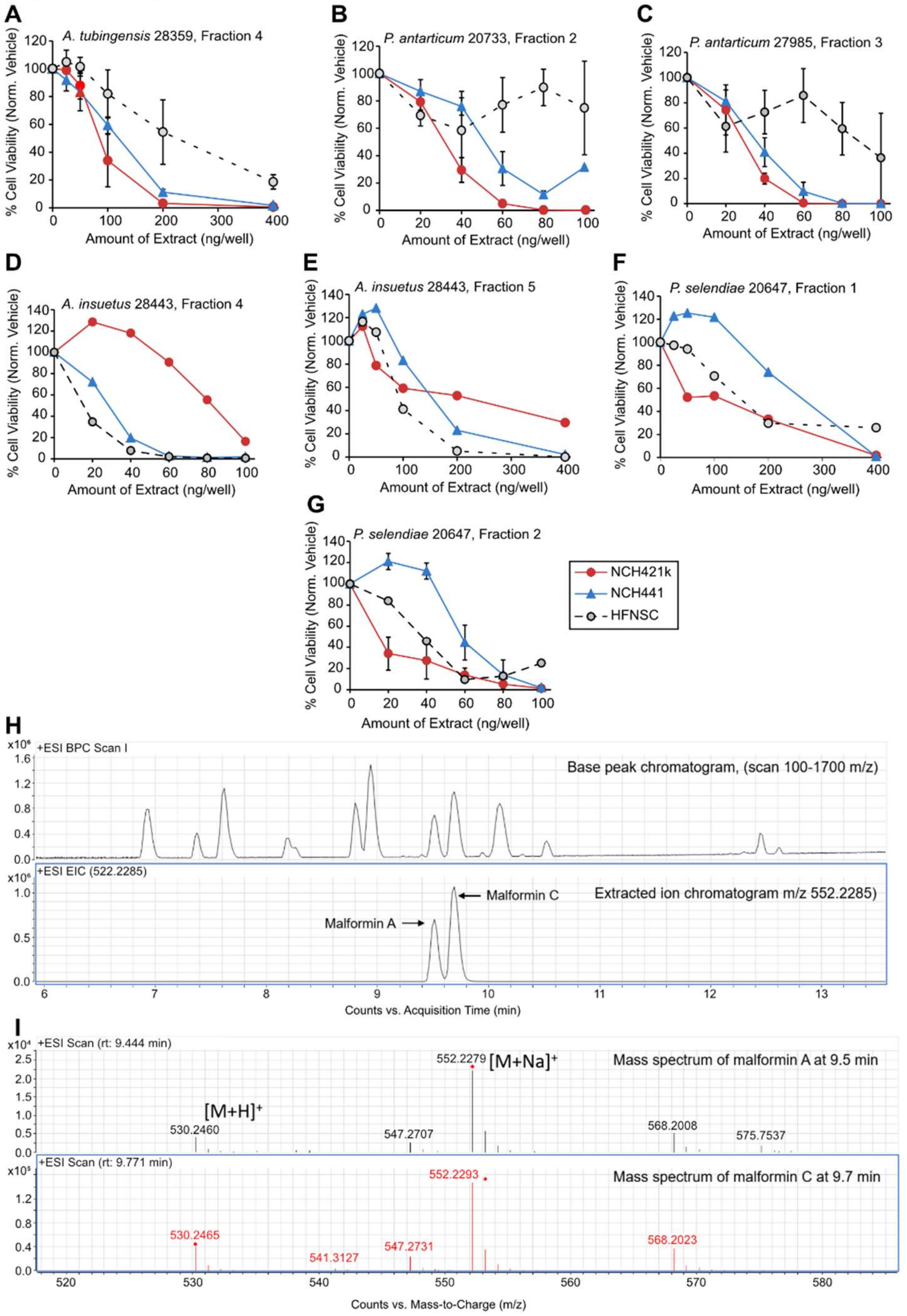

Active fractions tested in further cell lines and identification of malformin C

A-G) Normalized viability of NCH421k, NCH441 and human fetal neural stem cells (HFNSC) after 48 hours incubation with different amounts of the active fractions indicated in Figure S2. Extracts shown in A, B, C and G were tested in biological triplicate, error bars represent s.d.; D, E and F show an average of technical triplicates tested in a single biological replicate. **H)** Base peak chromatogram (BPC) of the initial hit fraction SK0604 generated from *Aspergillus tubingensis* (upper panel). Extracted ion chromatogram illustrating detection of both malformin A and C as their sodium adduct ($[M^{+}Na]^{+}$) at m/z 552.2285 Da, since both compounds have the same elemental composition ($C_{23}H_{39}N_5O_5S_2$) (lower panel). **I)** Mass spectra of malformin A and C, detected at 9.5 min and 9.7 min respectively

Supplementary Figure S3

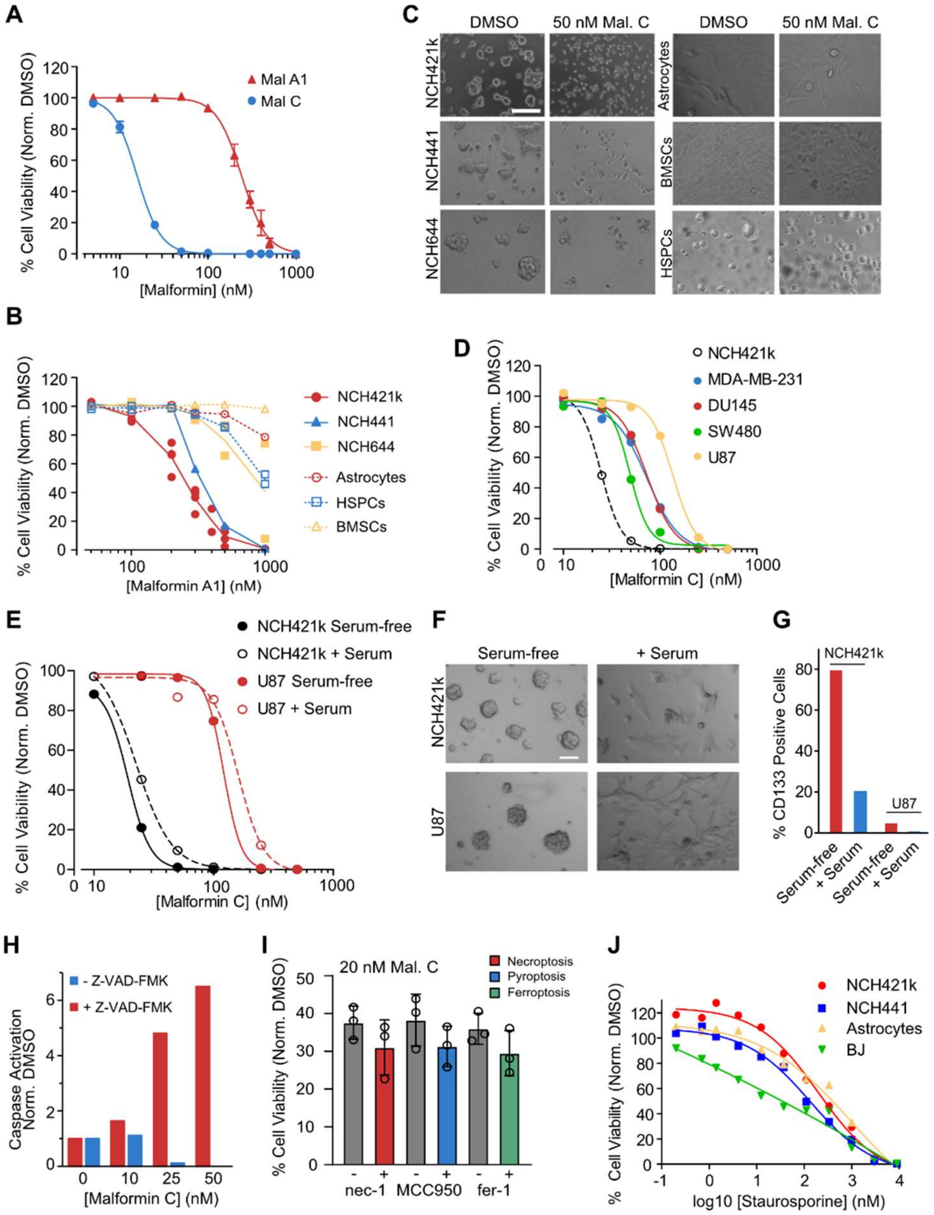

Characterization of cell death induced by the malformins

A) Cell viability of NCH421k glioblastoma stem-like cells (GSCs) incubated for 48 hours with increasing concentrations of malformins A1 and C (n=3, mean ±SD). **B)** Cell viability of NCH421k, NCH441 and NCH644 GSCs and normal cells (astrocytes, murine bone marrow stromal cells (BMSCs) and murine hematopoietic and progenitor cells (HPSCs) incubated for 48 hours with increasing concentrations of malformin A1 (n=3). **C)** Representative images of the cell lines in B and after incubation with 50 nM malformin C for 48 hours (scale bar = 50 µm) **D)** Cell viability of various cancer cell lines (MDA-MB-231, breast; DU145, pancreas; SW480, colon; U87, glioblastoma) incubated for 48 hours with increasing concentrations of malformin C, with NCH421k GSCs shown for comparison (n=1, mean of technical duplicates). **E)** Cell viability of NCH421k GSCs and U87 glioblastoma cells cultivated for 7 days with and without serum and then incubated for 48 hours with increasing concentrations of malformin C (n=1, mean of technical duplicates). **F)** Images of the adherent or spheroid growth phenotype of the cells from E (scale bar = 50 µm). **G)** CD133 staining showing differentiation status of cells from E and F (n=1). **H)** Caspase 3/7 activation of NCH421k GSCs incubated for 48 hours with malformin C, with or without 20 µM pan-caspase inhibitor Z-VAD-FMK (n=1, mean of

technical duplicates). **I)** Normalized viability of NCH421k GSCs incubated with or without 20 nM malformin C, and with or without 4 hours pre-treatment with 10 µM necrostatin (nec-1), 5 µM MCC950 or 20 µM ferrostatin (fer-1). Data are normalized to the respective DMSO control with or without inhibitor (set to 100 %, not shown). n=3, mean ± SD. **J)** Cell viability of NCH421k and NCH441 GSCs and astrocytes and human fibroblasts (BJ cells) incubated for 48 hours with increasing concentrations of staurosporine (n=1, mean of technical duplicates).

Supplementary Figure S4

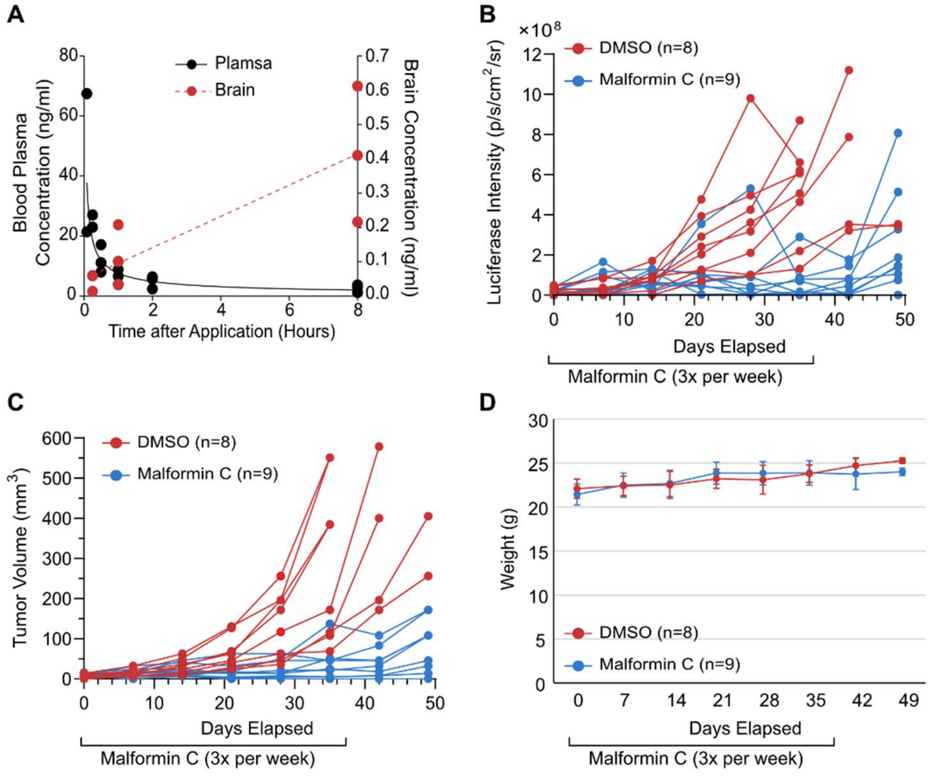

Malformin C slows tumor growth *in vivo*

A) Blood plasma concentration and brain concentration of malformin C in mice over 8 hours after *i.v.* injection with 0.5 mg/kg (n=3 mice per time point). **B)** Tumor size as determined by bioluminescence over 49 days in individual mice. **C)** Tumor as determined by caliper measurement over 49 days in individual mice. **D)** Body weight of the mice over the duration of the study.

Supplementary Figure S5

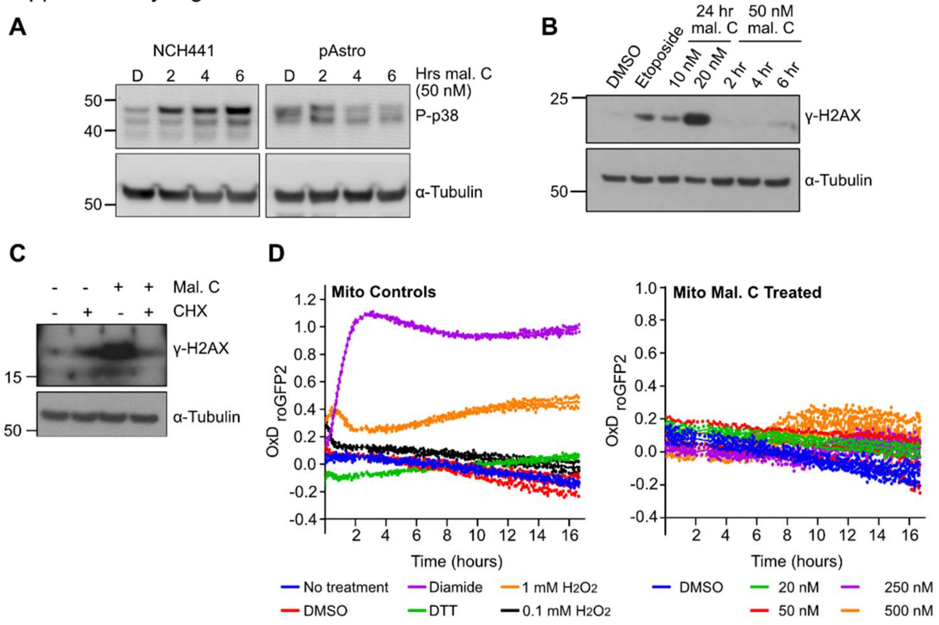

Investigation of malformin C-induced DNA damage and oxidative stress in glioblastoma stem-like cells

A) Western blot showing phosphorylated p38 levels in NCH441 glioblastoma stem-like cells (GSCs) and primary astrocytes over 6 hours incubation with 50 nM malformin C. α-tubulin is shown as a loading control. **B)** Western blot showing γ-H2AX levels in NCH421k GSCs with incubation of malformin C over 24 hours at 10, 20 and 50 nM. Etoposide (50 μM) was used as a positive control for inducing DNA damage. α-tubulin is shown as a loading control. **C)** Western blot showing γ-H2AX levels in NCH421k GSCs which were incubated with or without 20 nM malformin C for 24 hours, and with or with pre-treatment with cycloheximide (CHX) for 4 hours. α-tubulin is shown as a loading control. **D)** Mitochondrial roGFP2 oxidation of NCH421k GSCs with incubation of controls (H_2O_2 = positive control for ROS generation; diamide = fully oxidized sample; DTT = reduced sample) or increasing concentrations of malformin C over 16 hours, n=1.

Supplementary Figure S6

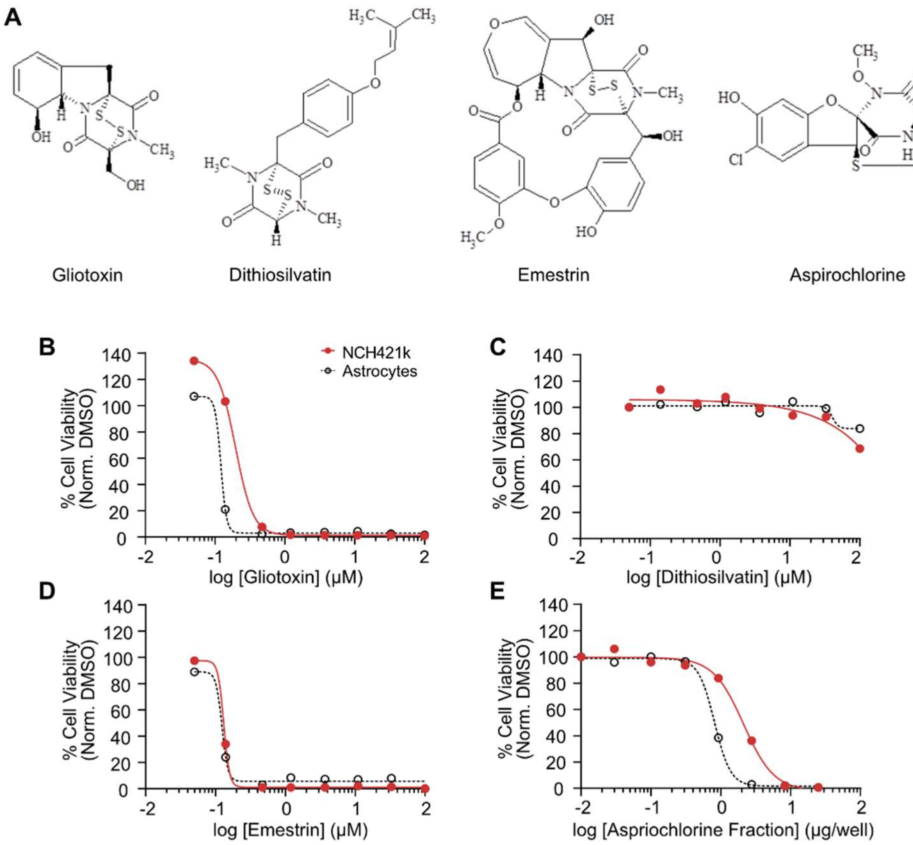

Glioblastoma stem-like cells are not sensitive to other compounds containing disulfide bonds

A) Structures of disulfide bond-containing gliotoxin, dithiosilvatin, emestrin, aspirochlorine. **B)** Normalized cell viability of NCH421k GSCs and primary astrocytes after incubation with increasing concentrations of each compound for 48 hours, n=1, mean of technical duplicates.

Supplementary Figure S7

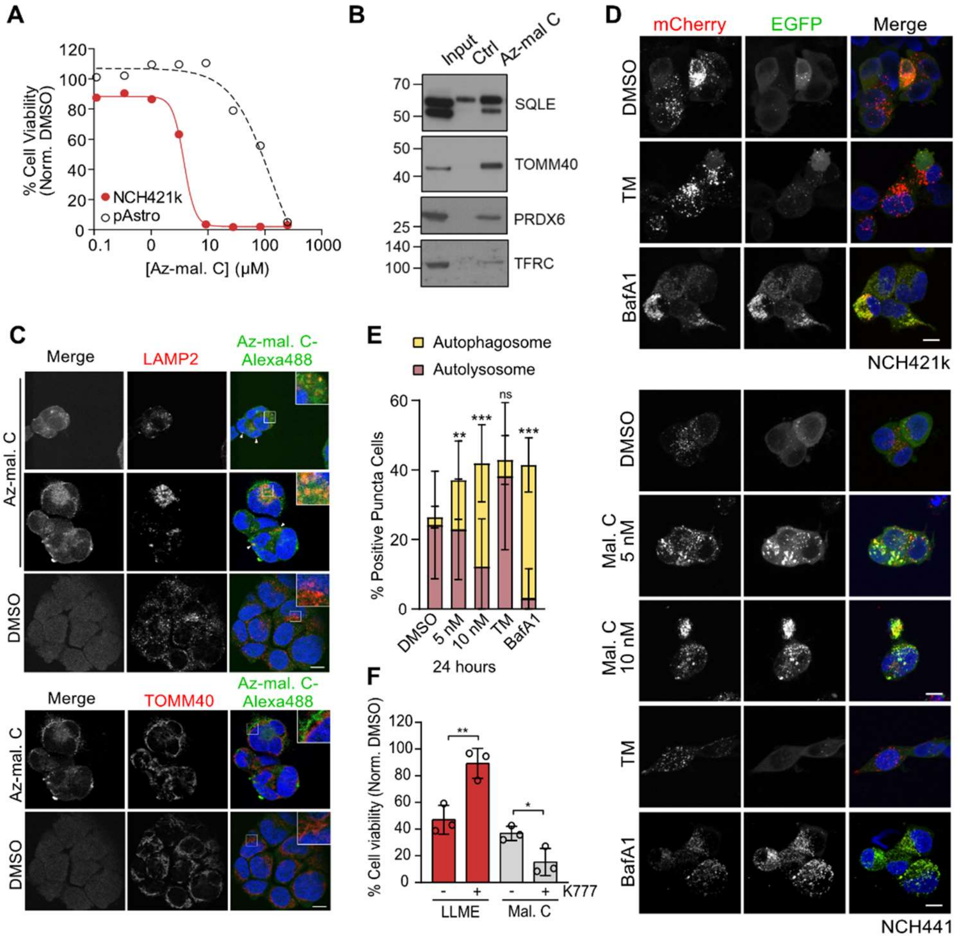

Malformin C disrupts autophagic flux in glioblastoma stem-like cells

A) Normalized viability of NCH421k glioblastoma stem-like cells (GSCs) and primary astrocytes after incubation with increasing concentrations of Az-mal. C for 48 hours. **B)** Confirmation of enrichment of SQLE, TOMM40, PRD6X and TFRC on Az-mal. C bound beads by Western blot. **C)** Confocal microscopy images of DMSO or Az-mal. C with covalently bound Alexa fluor 488-alkyne (green), along with immunostaining of lysosomal marker LAMP2 or mitochondrial marker TOMM40 (red), in NCH421k GSCs. Cells were incubated with 30 µM Az-mal. C for 3 hours with subsequent binding of Alexa-fluor-488. Scale bar = 10 µm. **D)** Representative images of NCH421k and NCH441 GSCs stably expressing mCherry-GFP-LC3B after incubation with 5 nM and 10 nM malformin C (NCH441 only), 1 µg/ml tunicamycin or 30 nM bafilomycin A1 for 24 hours. **E)** Quantification of % positive NCH441 GSCs for autolysosomes (red) or autophagosomes (yellow) after 24 hours incubation with 5 or 10 nM malformin C, 1 µg/ml tunicamycin or 30 nM bafilomycin A1. Data are representative of three (DMSO, malformin C) or two (TM, baf A1) independent experiments with 5 images taken for each experiment, mean ± SD, one-way ANOVA, *$p < 0.05$, **$p < 0.01$, ***$p < 0.001$, ns = not significant (numbers of autophagosomes compared with DMSO). **F)** Normalized viability of NCH421k GSCs incubated with or without 20 nM malformin C, or 1 mM LLME, and with or without 4 hours pre-treatment with K777. Data are normalized to the respective DMSO control with or without K777 (set to 100 %, not shown). n=3, mean ± SD, two-tailed t-test, *$p < 0.05$, **$p < 0.01$.
