# Supplementary material for: Malformin C preferentially kills glioblastoma stem‐like cells via concerted induction of proteotoxic stress and autophagic flux blockade
Source: Mol Oncol. 2024 Oct 27;19(3):785–807. doi: 10.1002/1878-0261.13756 (PMC11887673; doi:10.1002/1878-0261.13756)
Supplement: Supplementary file 2 — Table S1. Fungal strains used in the extract screen and their sources. Table S2. The top 500 deregulated genes after incubation of 50 nm malformin C after 2, 4 and 6 h. Table S3. Top positively and negatively enriched signatures from gene set enrichment profiling using the gene ontology collection. Table S4. Top 20 most enriched proteins identified in Az‐mal. C pulldown. Table S5. Top 10 ‘canonical pathways’ identified from the 300 most enriched proteins from the Az‐mal. C pulldown. Table S6. Top 5 ‘molecular functions’ identified from the 300 most enriched proteins from the Az‐mal. C pulldown. [file MOL2-19-785-s001.zip › mol213756-sup-0003-Tables.pdf]

Supplementary Tables

Table S1

Fungal strains used in the extract screen and their sources.

IBT Number	Species	Source
28368	*Penicillium chrysogenum*	Galathea v 318-1, sea water
28450	*Aspergillus sydowii*	Galathea v 29-12, sea water
28303	*Aspergillus tubingensis*	Fanø, sea water
28359	*Aspergillus tubingensis*	Galathea v 318-7, sea water
20733	*Penicillium antarticum*	Seaweed
27985	*Penicillium antarticum*	Bellevue 100 v 8-1, sea water
28361	*Stilbella fimetaria*	Fanø 1-2, sea water
28294	*Penicillium bialowiezense*	Ringkøbing fjord -1, fjord water
27968	*Penicillium bialowiezense*	Bellevue 500-2, sea water
28443	*Aspergillus insuetus*	Galathea v 29-6, sea water
28293	*Aspergillus versicolor (II)*	Fanø 1-16, sea water
20892	*Pencillium glabrum*	Seaweed
20647	*Pencillium selandiae*	Herring
27986	*Pencillium olsonii*	Bellevue, sea water
27975	*Aspergillus candidus*	Bellevue, sea water

Table S2

The top 500 deregulated genes after incubation of 50 nM malformin C after 2, 4 and 6 hours: provided in a separate Excel document.

Table S3

Top positively and negatively enriched signatures from gene set enrichment profiling using the gene ontology collection.

	NES	FWER p-val
**2 Hours**		
GO_PERK_MEDIATED_UNFOLDED_PROTEIN_RESPONSE	2.66	0.001
GO_R_SMAD_BINDING	2.54	0.009
GO_POSITIVE_REGULATION_OF_VASCULAR_SMOOTH_MUSCLE_CELL_PROLIFERATION	2.51	0.012
GO_ER_NUCLEUS_SIGNALING_PATHWAY	2.49	0.014
GO_POSITIVE_REGULATION_OF_PRI_MIRNA_TRANSCRIPTION_BY_RNA_POLYMERASE_II	2.43	0.038
GO_EPITHELIAL_CELL_FATE_COMMITMENT	-2.25	0.001
GO_NEPHRON_TUBULE_FORMATION	-2.22	0.002
GO_HEART_VALVE_FORMATION	-2.18	0.003
GO_METANEPHRIC_TUBULE_MORPHOGENESIS	-2.16	0.005
GO_SEGMENT_SPECIFICATION	-2.15	0.007
**4 Hours**		
GO_CELLULAR_RESPONSE_TO_EXTRACELLULAR_STIMULUS	2.46	0
GO_REGULATION_OF_DNA_TEMPLATED_TRANSCRIPTION_IN_RESPONSE_TO_STRESS	2.43	0
GO_RNA_POLYMERASE_II_ACTIVATING_TRANSCRIPTION_FACTOR_BINDING	2.41	0
GO_CELLULAR_RESPONSE_TO_EXTERNAL_STIMULUS	2.41	0
GO_ACTIVATING_TRANSCRIPTION_FACTOR_BINDING	2.40	0
GO_MITOCHONDRIAL_RESPIRATORY_CHAIN_COMPLEX_ASSEMBLY	-2.25	0.004
GO_CONDENSED_NUCLEAR_CHROMOSOME_CENTROMERIC_REGION	-2.20	0.009
GO_NADH_DEHYDROGENASE_COMPLEX_ASSEMBLY	-2.06	0.179
GO_REGULATION_OF_PHOTORECEPTOR_CELL_DIFFERENTIATION	-2.04	0.215
GO_MITOCHONDRIAL_ELECTRON_TRANSPORT_NADH_TO_UBIQUINONE	-2.04	0.22
**6 Hours**		
GO_PRI_MIRNA_TRANSCRIPTION_BY_RNA_POLYMERASE_II	2.32	0
GO_RNA_POLYMERASE_II_ACTIVATING_TRANSCRIPTION_FACTOR_BINDING	2.31	0
GO_ACTIVATING_TRANSCRIPTION_FACTOR_BINDING	2.30	0
GO_POSITIVE_REGULATION_OF_PRI_MIRNA_TRANSCRIPTION_BY_RNA_POLYMERASE_II	2.27	0
GO_P38MAPK_CASCADE	2.21	0.003
GO_TRANSLATIONAL_TERMINATION	-2.27	0
GO_MITOCHONDRIAL_TRANSLATION	-2.23	0
GO_MITOCHONDRIAL_TRANSLATIONAL_TERMINATION	-2.21	0
GO_MITOCHONDRIAL_MATRIX	-2.21	0
GO_TRANSLATIONAL_ELONGATION	-2.17	0

Table S4

Top 20 most enriched proteins identified in Az-mal. C pulldown.

Gene Symbol	Full Name	Upreg in GBM?*
XPO5	Exportin-5	no
ATXN10	Ataxin-10	no
CDK4	Cyclin-dependent kinase 4	yes
ENDOD1	Endonuclease domain-containing 1 protein	no
TOMM40	Mitochondrial import receptor subunit TOM40 homolog	no
NCAM1	Neural cell adhesion molecule 1	no
FAM134A	Protein FAM134A	no
TNPO3	Transportin-3	yes
PRAF2	PRA1 family protein 2	no
TFRC	Transferrin receptor protein 1	yes
ATP1A1	Sodium/potassium-transporting ATPase subunit alpha-1	yes**
TNPO2	Transportin-2	no
CD63	Tetraspanin;CD63 antigen	yes
SCAMP2	Secretory carrier-associated membrane protein 2	yes
IGF2R	Cation-independent mannose-6-phosphate receptor	yes
CYB5B	Cytochrome b5 type B	yes
LGALS1	Galectin-1	yes
SLC44A1	Choline transporter-like protein 1	yes
FAM134B	Protein FAM134B	no
LAMTOR1	Regulator complex protein LAMTOR1	yes

*On mRNA level according to GEPIA2 database (TCGA GB data vs GTEX normal brain tissue) (http://gepia2.cancer-pku.cn)

**mRNA not upregulated but protein reported to be highly expressed in GSCs

Table S5

Top 10 "canonical pathways" identified from the 300 most enriched proteins from the Az-mal. C pulldown (Ingenuity Pathway Analysis).

Pathway	Molecules
RAN Signaling	CSE1L, IPO5, KPNA2, KPNB1, TNPO1, XPO1
Cholesterol Biosynthesis I	DHCR7, FDFT1, MSMO1, SQLE
Cholesterol Biosynthesis II (via 24,25-dihydrolanosterol)	DHCR7, FDFT1, MSMO1, SQLE
Cholesterol Biosynthesis III (via Desmosterol)	DHCR7, FDFT1, MSMO1, SQLE
Superpathway of Cholesterol Biosynthesis	DHCR7, FDFT1, HMGCS1, MSMO1, SQLE
CLEAR Signaling Pathway	ATP6V1H, CD63, HRAS, IGF2R, LAMTOR1, M6PR, MAPK3, PDGFRA, PPP2R1A, PPP2R2A, PPP2R5A, PPP2R5E, YWHAH
Tight Junction Signaling	CDK4, CSTF2, CTNNA1, JAM3, PPP2R1A, PPP2R2A, PPP2R5A, PPP2R5E, SYMPK, VAPA
Regulation of eIF4 and p70S6K Signaling	EIF3K, EIF3L, HRAS, ITGB5, MAPK3, PPP2R1A, PPP2R2A, PPP2R5A, PPP2R5E ,RPS12
Cell Cycle Regulation by BTG Family Proteins	CDK4, PPP2R1A, PPP2R2A, PPP2R5A, PPP2R5E
Epoxysqualene Biosynthesis	FDFT1, SQLE

Table S6

Top 5 "molecular functions" identified from the 300 most enriched proteins from the Az-mal. C pulldown (Ingenuity Pathway Analysis).

Pathway	Number of Molecules
Molecular Transport	99
Protein Trafficking	36
Cell Death and Survival	133
Cellular Compromise	66
Cellular Assembly And Organization	88
